# Which Clinical, Radiological, Inflammatory, and Lesion-Related Characteristics Are Associated with Early Neurological Decompensation After Intracranial Mass Lesion Surgery?

**DOI:** 10.3390/diagnostics16193240

**Published:** 2026-10-07

**Authors:** Fatma Betül Saylak, Burak Bahadır

**Affiliations:** 1 Department of Neurosurgery, Çankırı State Hospital, Çankırı 18100, Türkiye; 2 Department of Neurosurgery, Ankara Bilkent City Hospital, Ankara 06800, Türkiye

**Keywords:** intracranial mass lesions, early neurological decompensation, Karnofsky Performance Status, neutrophil-to-lymphocyte ratio, postoperative neurological outcomes

## Abstract

**Background:** Early neurological decompensation is a clinically relevant concern following intracranial surgery, but factors associated with this early outcome remain incompletely characterized. This study evaluated factors associated with neurological decompensation within 48 h after surgery. **Methods:** This retrospective, single-center study included 301 surgical episodes involving intracranial mass lesions. Early neurological decompensation was defined as a ≥2-point decrease in Glasgow Coma Scale (GCS) score, the development of a new neurological deficit, or the objective worsening of a pre-existing deficit within 48 h. Clinical, radiological, lesion-related, and hematological factors were evaluated using logistic regression and ROC analyses, with bootstrap internal validation. **Results:** Early neurological decompensation occurred in 63 surgical episodes (20.9%). Preoperative Karnofsky Performance Status (KPS) was independently associated with the outcome (aOR = 0.50; 95% CI: 0.38–0.67 per 10-point increase; corresponding per-1-point aOR = 0.93; 95% CI: 0.91–0.96; *p* < 0.001), while peritumoral edema showed a statistically significant but imprecisely estimated association (aOR = 5.10; 95% CI: 1.14–22.79; *p* = 0.033). In extended models, malignant lesion category and peripheral contrast enhancement were independently associated with the outcome, whereas NLR was not after multivariable adjustment. The primary model had an AUC of 0.793 and a bootstrap-corrected AUC of 0.777. **Conclusions:** Preoperative KPS showed the most consistent independent association with early neurological decompensation. Additional radiological and lesion-related factors did not demonstrate significant incremental discrimination beyond KPS alone. External validation is required before clinical application.

## 1. Introduction

Surgical resection remains a cornerstone of treatment for intracranial mass lesions, with contemporary neurosurgical practice seeking to achieve the maximal extent of safe resection while preserving neurological function [1]. Despite advances in surgical planning, intraoperative neuromonitoring, and functional mapping, postoperative neurological deterioration continues to represent a clinically relevant complication of intracranial mass lesion surgery [2]. Recent studies have demonstrated that postoperative neurological deficits may compromise functional recovery and influence subsequent clinical outcomes, emphasizing preservation of neurological function as an integral component of surgical success [3]. Identifying patients at increased risk of early postoperative neurological deterioration is therefore important for refining perioperative risk assessment and tailoring the intensity of postoperative surveillance to individual patient needs [4].

Preoperative functional status represents a key clinical dimension in anticipating the early neurological course after surgery and may provide insight into a patient’s functional reserve before exposure to surgical stress [5]. The Karnofsky Performance Status (KPS), in particular, provides a standardized measure of overall functional capacity in patients with intracranial mass lesions, and poorer preoperative performance has been linked to less favorable postoperative outcomes [6]. Neurological risk, however, is unlikely to be determined by baseline functional capacity alone. Radiological characteristics—including lesion size and location, peritumoral edema, and mass effect—may also shape neurological vulnerability during the postoperative period [7]. The heterogeneity of postoperative neurological trajectories among patients with otherwise comparable clinical profiles therefore highlights the need to investigate additional factors that may be associated with early postoperative neurological outcomes alongside conventional measures of functional status and radiological lesion characteristics [8].

Beyond clinical and radiological risk factors, increasing attention has been directed toward the contribution of the systemic inflammatory response arising from tumor–host interactions to neuro-oncological outcomes [9]. In this context, peripheral blood-derived indices, including the neutrophil-to-lymphocyte ratio (NLR) and systemic immune-inflammation index (SII), have been investigated as prognostic biomarkers. In patients with glioblastoma in particular, systemic inflammatory markers—most notably NLR—have been reported to be associated with survival [9]. Evidence regarding the prognostic relevance of SII has likewise focused predominantly on long-term oncological endpoints, such as overall and progression-free survival [10]. In contrast, the relationship of NLR and SII with early neurological deterioration following intracranial tumor surgery remains insufficiently characterized, as does the extent to which these indices provide information beyond established clinical, functional, and radiological factors.

The present study evaluated clinical, radiological, lesion-related, and systemic inflammatory factors associated with early neurological decompensation following surgery for intracranial mass lesions. We examined the relationships of preoperative functional status, radiological and lesion-related characteristics, and systemic inflammatory markers with the early postoperative neurological course. We further assessed these factors in multivariable analyses to determine which were independently associated with early neurological decompensation and whether inflammatory indices provided additional information beyond clinical and radiological factors.

## 2. Materials and Methods

### 2.1. Study Design and Patient Population

**Figure 1:**
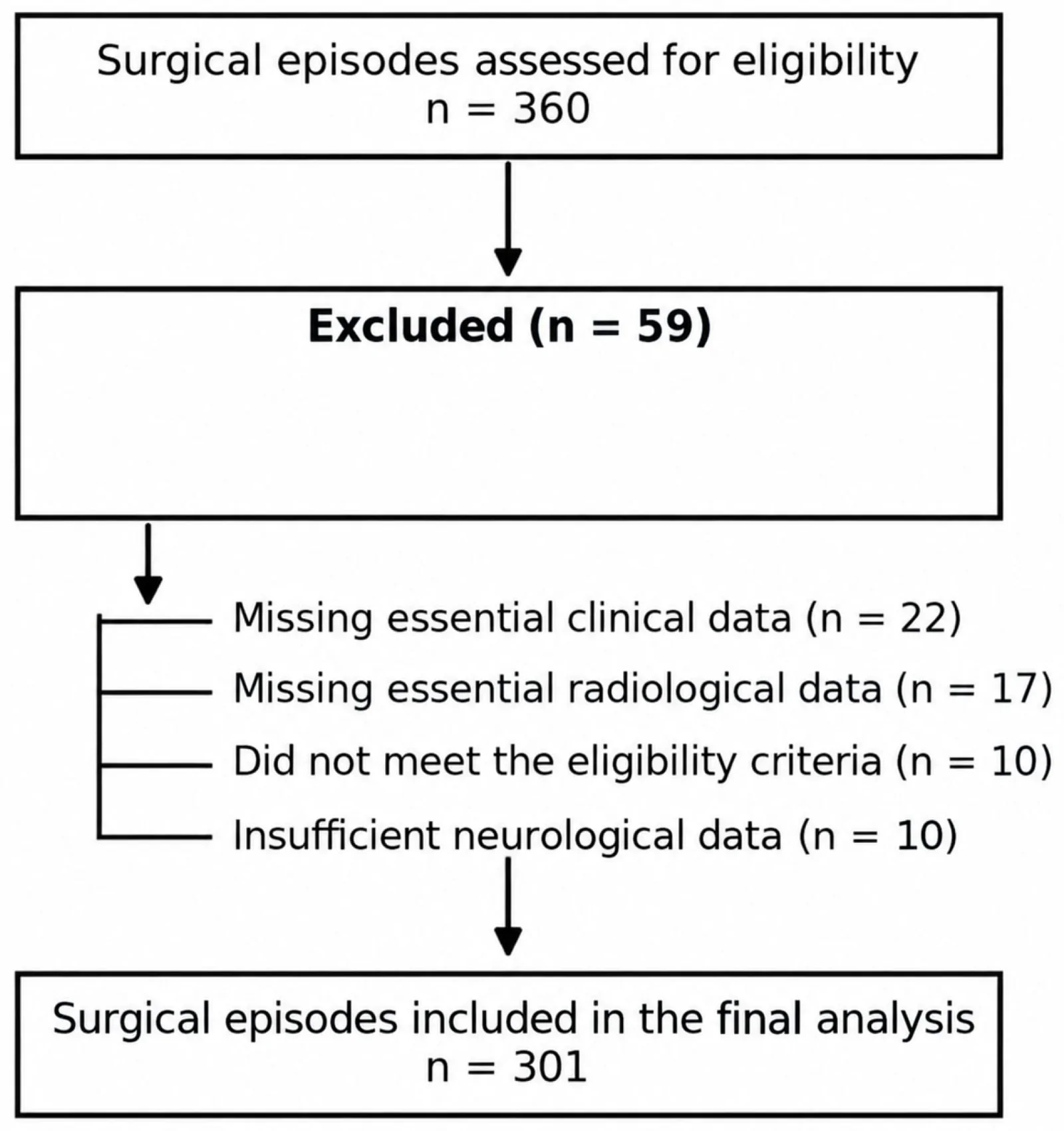
Study flow diagram showing the 360 surgical episodes assessed for eligibility, reasons for exclusion (n = 59), and the final analytic cohort of 301 surgical episodes corresponding to 293 unique patients.

We retrospectively evaluated patients aged ≥18 years who underwent surgical treatment for an intracranial mass lesion at the Department of Neurosurgery, Ankara Bilkent City Hospital, between 1 April 2019, and 1 January 2026. The study was conducted as a single-center retrospective cohort investigation. The study was reported in accordance with the TRIPOD 2015 statement, and the completed checklist is provided as Supplementary File S1. The source population was restricted to cases managed by two neurosurgeons during the study period and therefore did not represent the entire intracranial mass lesion surgery volume of the department. During the study period, 360 surgical cases were assessed for eligibility. Of these, 59 were excluded because of missing essential clinical data (n = 22), missing essential radiological data (n = 17), failure to meet the eligibility criteria (n = 10), or insufficient neurological data (n = 10). The primary analysis therefore included 301 surgical episodes corresponding to 293 unique patients (Figure 1). Eight patients underwent two qualifying operations during the study period and consequently contributed two surgical episodes each. Among these eight patients, the interval between the first and second operations was 6 months in five patients and 9 months in three patients (median, 6 months; range, 6–9 months). All repeat operations were performed for recurrent disease and were not related to early neurological decompensation following the first procedure. The surgical episode was defined as the unit of analysis for the primary analysis. To assess the potential influence of repeated observations, a sensitivity analysis restricted to one surgical episode per patient (N = 293) was also performed.

Eligibility required surgical treatment for an intracranial mass lesion, together with the availability of the preoperative and early postoperative neurological data necessary to ascertain the primary endpoint. Patients were excluded when the available records did not permit evaluation of the primary outcome or key study variables.

Early neurological decompensation occurring within the first 48 h after surgery was designated as the primary endpoint. Because repeated surgical procedures in the same patient could potentially influence the findings, a separate sensitivity analysis was undertaken after removing observations related to repeat operations.

The study protocol was approved by the Clinical Research Ethics Committee of Ankara Bilkent City Hospital (Decision No. TABED 2-26-2731; 16 September 2026). Retrospective data extraction and statistical analyses for the present study were initiated only after ethics committee approval had been obtained. The study was conducted in accordance with the principles of the Declaration of Helsinki.

### 2.2. Clinical and Perioperative Variables

Demographic, clinical, and perioperative data were retrospectively retrieved from electronic medical records, patient charts, and operative documentation. The demographic and clinical variables comprised age, sex, and the presence of comorbidities. Comorbidity was coded as a binary present/absent variable derived from the free-text comorbidity/problem-list entries in the medical record. Patients were classified as comorbidity-present if at least one documented condition other than an explicit “none” entry was recorded, and comorbidity-absent otherwise (204/301, 67.8% present). Conditions captured under this variable included hypertension, diabetes mellitus, coronary artery disease/other cardiac disease, prior cerebrovascular accident/stroke, dementia, chronic obstructive pulmonary disease, hypothyroidism, cardiac arrhythmia, and congestive heart failure. This composite present/absent coding did not correspond to a validated comorbidity index (e.g., Charlson Comorbidity Index or ASA class).

Neurological status was evaluated using the Glasgow Coma Scale (GCS), whereas functional status was assessed using the standard 0–100 Karnofsky Performance Status (KPS) scale. Preoperative GCS and KPS scores were obtained from the last documented clinical assessment before surgery. KPS assessments documented in the medical records were performed by a single neurosurgeon, who is a co-author of the present study, as part of routine preoperative clinical evaluation. Postoperative neurological assessments were performed within the first 48 h after surgery by the same neurosurgeon who performed the preoperative KPS assessments. The examiner was not blinded to the preoperative neurological status. Motor function was assessed using the Medical Research Council (MRC) scale. These assessments were part of routine postoperative clinical care rather than study-specific evaluations.

Surgical and perioperative variables comprised operative duration, extent of resection, and the use of intraoperative neuromonitoring. Extent of resection was categorized as total resection, subtotal resection, or biopsy. Extent of resection was determined on the basis of early postoperative imaging rather than solely from the surgeon’s intraoperative impression; no formal volumetric segmentation was performed.

### 2.3. Radiological and Lesion-Related Assessment

Preoperative radiological characteristics were evaluated using preoperative cranial magnetic resonance imaging and/or computed tomography studies. The radiological variables comprised lesion area, midline shift, the presence of peritumoral edema, and contrast-enhancement pattern. Preoperative images were evaluated by the same neurosurgeon who performed the clinical assessments described above; this assessor performed the radiological measurements and determined the presence or absence of the radiological features included in the analysis. Early postoperative cranial CT was performed within the first 6 h after all surgical procedures to assess the extent of resection.

Lesion area was estimated in cm^2^ by multiplying the two largest perpendicular lesion diameters on preoperative imaging. In patients with multiple intracranial lesions, the largest surgically treated lesion served as the reference lesion for analysis. Midline shift was recorded in millimeters. Peritumoral edema was assessed on preoperative MRI and was defined as T2/FLAIR hyperintensity surrounding the lesion. Edema was recorded dichotomously as present or absent because quantitative or volumetric edema measurements were not included in the retrospective dataset.

All surgical procedures included in the study were elective, defined as procedures performed on a scheduled, non-emergency basis, and were performed through a transcranial craniotomy approach. The small number of abscesses and hematomas included in the cohort required surgical treatment but were managed on a scheduled, non-emergency basis. Of the 301 surgical episodes, 266 (88.4%) involved supratentorial lesions and 35 (11.6%) involved infratentorial/posterior fossa lesions. Lesion location was categorized as frontal, temporal, parietal, frontoparietal, occipital, cerebellar/posterior fossa, or other/deep location. The other/deep category comprised less frequent locations, including skull-base, intraventricular, frontotemporal, corpus callosum/thalamic, and temporo-occipital lesions, which were pooled because of the small numbers of surgical episodes and outcome events within the individual categories. A formal classification of lesion eloquence, such as the Sawaya grading system, was not available in the retrospective dataset. Contrast-enhancement pattern was categorized as homogeneous, heterogeneous, peripheral, or absent.

Histopathological diagnoses were extracted from the final pathology reports, and lesions were classified into four categories: metastatic tumors (n = 105), primary malignant central nervous system (CNS) tumors (n = 68), primary benign/low-grade CNS tumors (n = 102), and non-neoplastic lesions (n = 26). The non-neoplastic category comprised gliosis (n = 18), abscess (n = 4), hematoma (n = 2), and necrosis (n = 2). Five lesions initially classified as indeterminate were reclassified as metastatic tumors based on definitive histopathological findings consistent with metastatic disease in conjunction with a documented extracranial primary malignancy identified either at the time of surgery or during subsequent oncological work-up. The 18 gliosis cases represented lesions that were radiologically suspicious for a neoplastic mass because of their mass-like appearance with associated edema and/or contrast enhancement on preoperative imaging and were therefore surgically treated as presumptive mass lesions; however, final histopathological examination demonstrated reactive/gliotic tissue without evidence of a neoplastic process.

For multivariable analyses, these four lesion categories were additionally consolidated into two groups to evaluate the association between lesion category and early neurological decompensation while reducing the risk of model overfitting associated with the limited number of outcome events. Metastatic tumors and primary malignant CNS tumors were classified as malignant (n = 173), whereas primary benign/low-grade CNS tumors and non-neoplastic lesions were classified as non-malignant (n = 128). This binary classification was used in the secondary extended model to examine whether lesion category provided additional explanatory information beyond the variables included in the primary model.

### 2.4. Laboratory and Systemic Inflammatory Markers

Hematological data were retrospectively retrieved from the hospital electronic medical record system. Preoperative hemoglobin (g/dL), platelet (×10^9^/L), neutrophil (×10^9^/L), and lymphocyte (×10^9^/L) levels were obtained from routine complete blood counts performed within 24 h before surgery. All patients received preoperative dexamethasone (8 mg) as part of routine clinical management; therefore, the preoperative complete blood count measurements used in the present analysis were obtained under corticosteroid exposure. Postoperative hematological measurements were derived from routine blood samples collected within the first 6 h after completion of surgery. These postoperative measurements and the derived perioperative change (Δ) variables were evaluated as exploratory perioperative variables rather than as baseline prognostic predictors, given their potential temporal overlap with the 48-h primary outcome.

Preoperative neutrophil-to-lymphocyte ratio (NLR) and systemic immune-inflammation index (SII) were calculated from the corresponding preoperative complete blood count parameters. Perioperative changes in hemoglobin, platelet, neutrophil, and lymphocyte values were additionally quantified by subtracting the preoperative measurement from the postoperative measurement (Δ = postoperative − preoperative).

### 2.5. Definition and Ascertainment of Early Neurological Decompensation

Early neurological decompensation within the first 48 h after surgery served as the primary endpoint. An event was considered to have occurred when at least one of the following criteria was met: (1) a postoperative decrease of ≥2 points from the preoperative Glasgow Coma Scale (GCS) score or (2) an objective postoperative neurological deficit, comprising either a newly developed neurological deficit or objective worsening of a pre-existing neurological deficit.

Motor strength was assessed as part of routine clinical care using the Medical Research Council (MRC) scale. For the present retrospective analysis, motor deficits were classified according to their documented presence or absence; patient-level preoperative-to-postoperative changes in MRC grade were not extracted as a structured quantitative variable. In patients with a pre-existing neurological deficit, postoperative worsening was determined clinically by comparison of the postoperative neurological examination with the documented preoperative examination. Thus, worsening of a pre-existing deficit was not treated as a separate third endpoint component but was classified within the objective neurological deficit component.

Preoperative and postoperative GCS scores and neurological examination findings were routinely documented in the hospital medical record system. Postoperative neurological assessment within the 48-h outcome window was performed by a single neurosurgeon and was not blinded to preoperative neurological status; postoperative findings were assessed with reference to the documented preoperative neurological examination to identify either a newly developed neurological deficit or objective worsening of a pre-existing deficit. No additional study-specific neurological assessment was performed for the purposes of this retrospective study; the analysis was based on routinely documented clinical data. Patients who met neither criterion were categorized as having no early neurological decompensation.

### 2.6. Statistical Analysis

Distributional characteristics of continuous variables were examined with the Shapiro–Wilk test. Depending on their distribution, continuous data are reported as mean ± standard deviation or median (interquartile range [IQR]). Between-group differences were examined using the independent-samples *t* test, with Welch’s correction when appropriate, or the Mann–Whitney U test. Categorical data are presented as n (%) and were compared using the chi-square test or Fisher’s exact test. Within-patient changes in preoperative and postoperative GCS, KPS, and hematological parameters were examined with the Wilcoxon signed-rank test. Relationships between continuous variables were quantified using Spearman’s rank correlation coefficient (ρ). No missing values were present for any variable included in the analyses reported here; consequently, all analyses used complete cases and no imputation procedure was required. Because perioperative change scores may depend on baseline values, additional analyses examined Δneutrophil and Δlymphocyte after adjustment for their corresponding preoperative values.

Factors associated with early neurological decompensation were initially examined by univariable binary logistic regression, with effect estimates reported as odds ratios (ORs) and 95% confidence intervals (CIs). For multivariable analysis, a parsimonious primary model (Model 1) comprising age, preoperative KPS, peritumoral edema, NLR, and comorbidity was specified with consideration of the number of outcome events (n = 63) and the events-per-variable ratio. Preoperative KPS was modeled as a continuous variable, with the OR per 1-point increase reported in the regression tables. Because KPS is conventionally scored in 10-point increments, the corresponding OR per 10-point increase was additionally calculated from the same fitted model and reported in the text to facilitate clinical interpretation.Candidate predictors for Model 1 were selected based on clinical relevance together with findings from univariable analysis, without threshold-based screening; preoperative GCS was not additionally entered because it overlaps conceptually with preoperative KPS as a measure of baseline neurological/functional status and also contributes directly to one component of the outcome definition; its simultaneous inclusion was therefore avoided to limit redundancy in the parsimonious primary model.

Two extended models were constructed to examine whether the primary findings persisted after accounting for lesion-related characteristics. Model 2 incorporated lesion category as a binary variable (malignant vs. non-malignant), with metastatic and primary malignant CNS tumors grouped as malignant and all remaining categories grouped as non-malignant. Model 3 incorporated the four-level contrast-enhancement pattern. Because no early neurological decompensation events occurred among non-enhancing lesions, resulting in separation, Model 3 was estimated using Firth penalized logistic regression. A Firth regression robustness analysis was also applied to Model 1 to examine potential estimation bias related to the limited number of events. Multicollinearity was assessed using the variance inflation factor (VIF). Firth’s penalized-likelihood logistic regression was implemented in Python 3.11 using the statsmodels (v0.15.0) and firthlogist packages; 95% confidence intervals for the Firth models were derived from profile-likelihood limits rather than Wald approximations, which is more appropriate given the small number of events in some strata. Model discrimination was quantified by the area under the receiver operating characteristic curve (AUC), and exploratory cutoff values were identified using the Youden index (pandas, scikit-learn, and SciPy). AUCs for preoperative KPS, KPS combined with peritumoral edema, and Model 1 were compared using the DeLong test.

Calibration of Model 1 was examined using the calibration slope, calibration intercept, and Brier score. Internal validation was performed with 1000 bootstrap resamples using Harrell’s optimism-correction method to estimate the optimism-corrected AUC and calibration slope. For graphical assessment of calibration, a LOESS-smoothed calibration curve with a bootstrap-derived 95% confidence band based on 2000 resamples was constructed. As a sensitivity analysis, Model 1 was re-estimated after excluding second surgical observations attributable to repeat operations. An additional sensitivity analysis was performed by re-estimating Model 1 after excluding non-neoplastic lesions to assess the consistency of the primary findings in the neoplastic-only subgroup. Model 1 was also re-estimated with anatomical location included as a categorical covariate, using cerebellar/posterior fossa as the reference category, to assess the robustness of the primary associations after adjustment for lesion location. The linearity of the logit for continuous predictors (age, preoperative KPS, NLR) was examined by adding quadratic terms to univariable models. Because evidence of nonlinearity was observed for preoperative KPS, additional sensitivity analyses were performed by modeling KPS categorically (≤70, 80, and 90–100) and using a restricted cubic spline with three degrees of freedom. The 95% CIs for AUC, sensitivity, and specificity were obtained by bootstrap resampling (2000 resamples, percentile method). All statistical tests were two-sided, with *p* < 0.05 considered statistically significant. The analysis code used for the peer-review sensitivity analyses is provided as Supplementary File S2.

## 3. Results

### 3.1. Sample Characteristics and Early Neurological Decompensation

**Figure 2:**
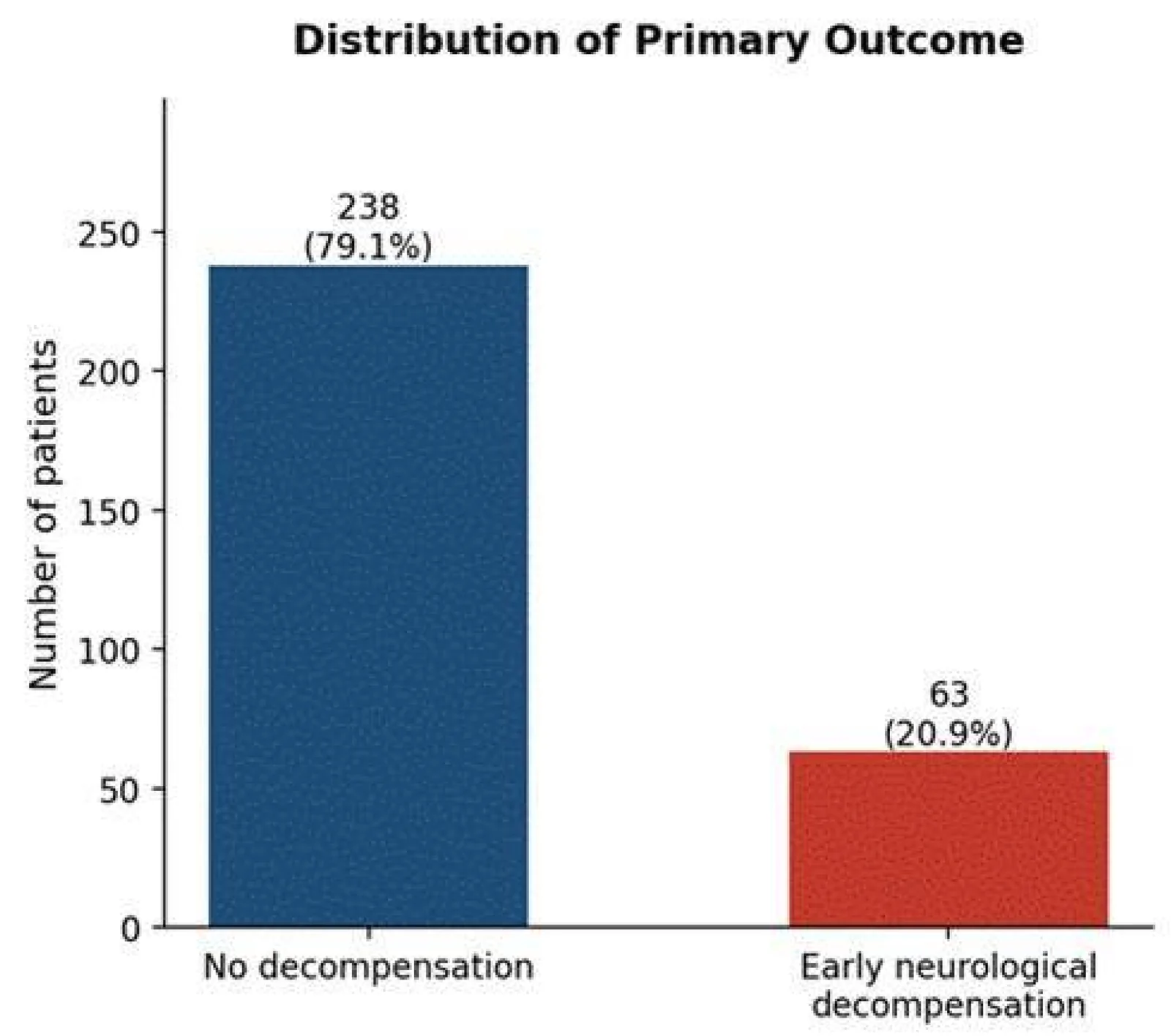
Distribution of the primary outcome (early neurological decompensation) across the full cohort.

The analysis comprised 301 surgical episodes, of which 63 (20.9%) met the criteria for early neurological decompensation (Figure 2). Of these, 58 (92.1%) met the objective neurological deficit criterion alone and 5 (7.9%) met the GCS decline criterion alone; no surgical episode met both criteria. Among the 58 surgical episodes meeting the objective neurological deficit criterion, 8 (13.8%) developed a new neurological deficit, whereas 50 (86.2%) experienced objective worsening of a pre-existing neurological deficit. The neurological deficit was motor in 51 episodes (87.9%), involved the facial nerve in 4 (6.9%), consisted of aphasia in 2 (3.4%), and involved the sixth cranial nerve in 1 (1.7%). At baseline, a focal neurological deficit was present in 59 of 301 surgical episodes (19.6%); 50 of these episodes were subsequently classified as early neurological decompensation events and 9 as non-events. Among the 242 episodes without a baseline focal neurological deficit, 13 were classified as events and 229 as non-events. Among the 63 surgical episodes meeting the primary endpoint, no postoperative hematoma or ischemic lesion was identified on early postoperative cranial CT performed within the first 6 h after surgery (0/63 for each).

Patients who experienced early neurological decompensation were older than those who did not (median, 68.0 vs. 61.0 years; *p* = 0.001) and had lower preoperative KPS scores (median, 80 vs. 90; *p* < 0.001). Preoperative GCS scores also differed significantly between the groups (median, 15 [IQR, 14–15] vs. 15 [IQR, 15–15]; *p* = 0.002). Peritumoral edema was more frequent among patients with early neurological decompensation (96.8% vs. 81.1%; *p* = 0.001) (Table 1).

### 3.2. Perioperative Changes in Laboratory and Clinical Measures

**Figure 3:**
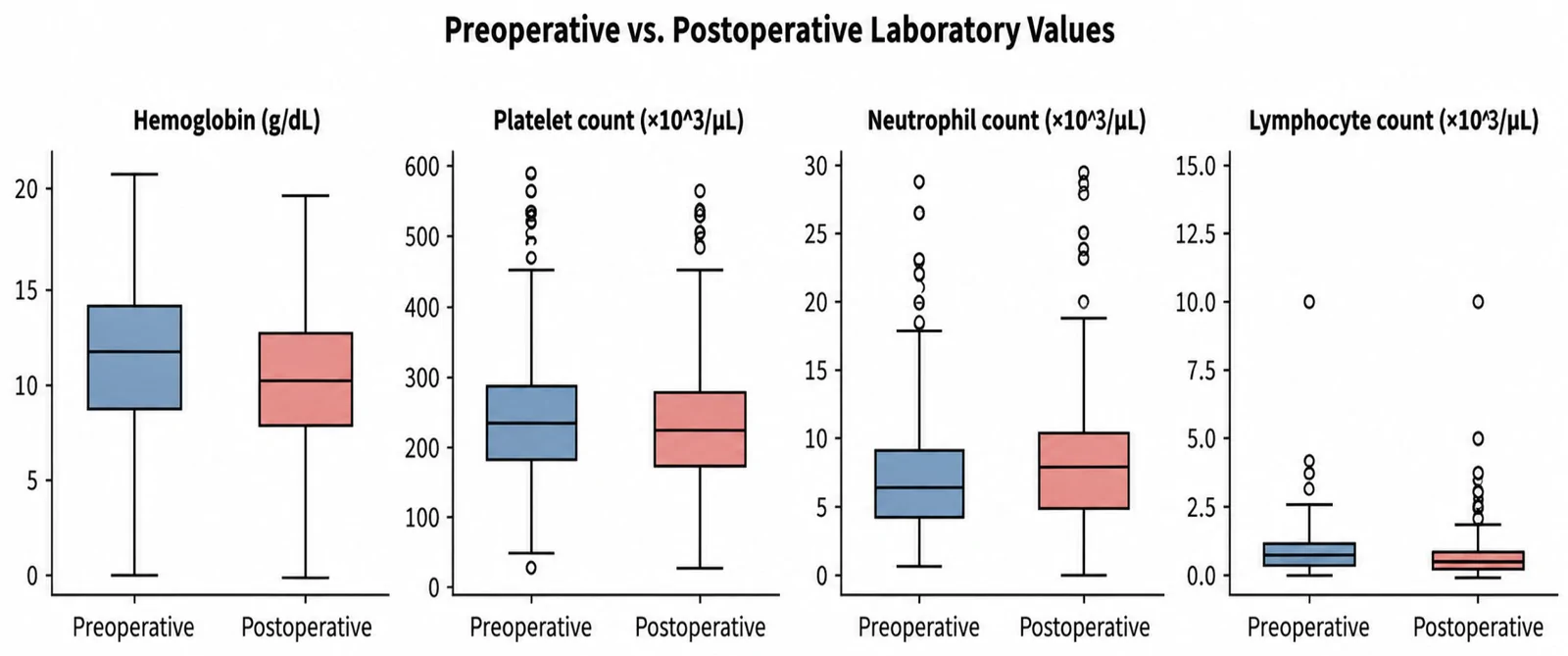
Preoperative versus postoperative hemoglobin, platelet, neutrophil, and lymphocyte values.

Within-episode comparisons demonstrated statistically significant perioperative changes across all six paired variables examined (Wilcoxon signed-rank test, all *p* < 0.05; Table 2, Figure 3). Postoperatively, hemoglobin decreased by a median of 0.60 g/dL (median Δ = −0.60 g/dL, *p* < 0.001), platelet count by 11.0 × 10^9^/L (median Δ = −11.0 × 10^9^/L, *p* < 0.001), and lymphocyte count by 0.15 × 10^9^/L (median Δ = −0.15 × 10^9^/L, *p* < 0.001). In contrast, neutrophil count increased (median Δ = +1.27 × 10^9^/L, *p* < 0.001). Although the median GCS remained 15.0 before and after surgery, individual patients showed paired changes in GCS, resulting in a statistically significant Wilcoxon signed-rank test (*p* = 0.010). Similarly, the median change in KPS was 0 despite a statistically significant paired difference (*p* < 0.001). Thus, these statistical differences reflect individual patient-level changes rather than a clinically meaningful overall improvement or deterioration.

### 3.3. Correlation Analyses

**Figure 4:**
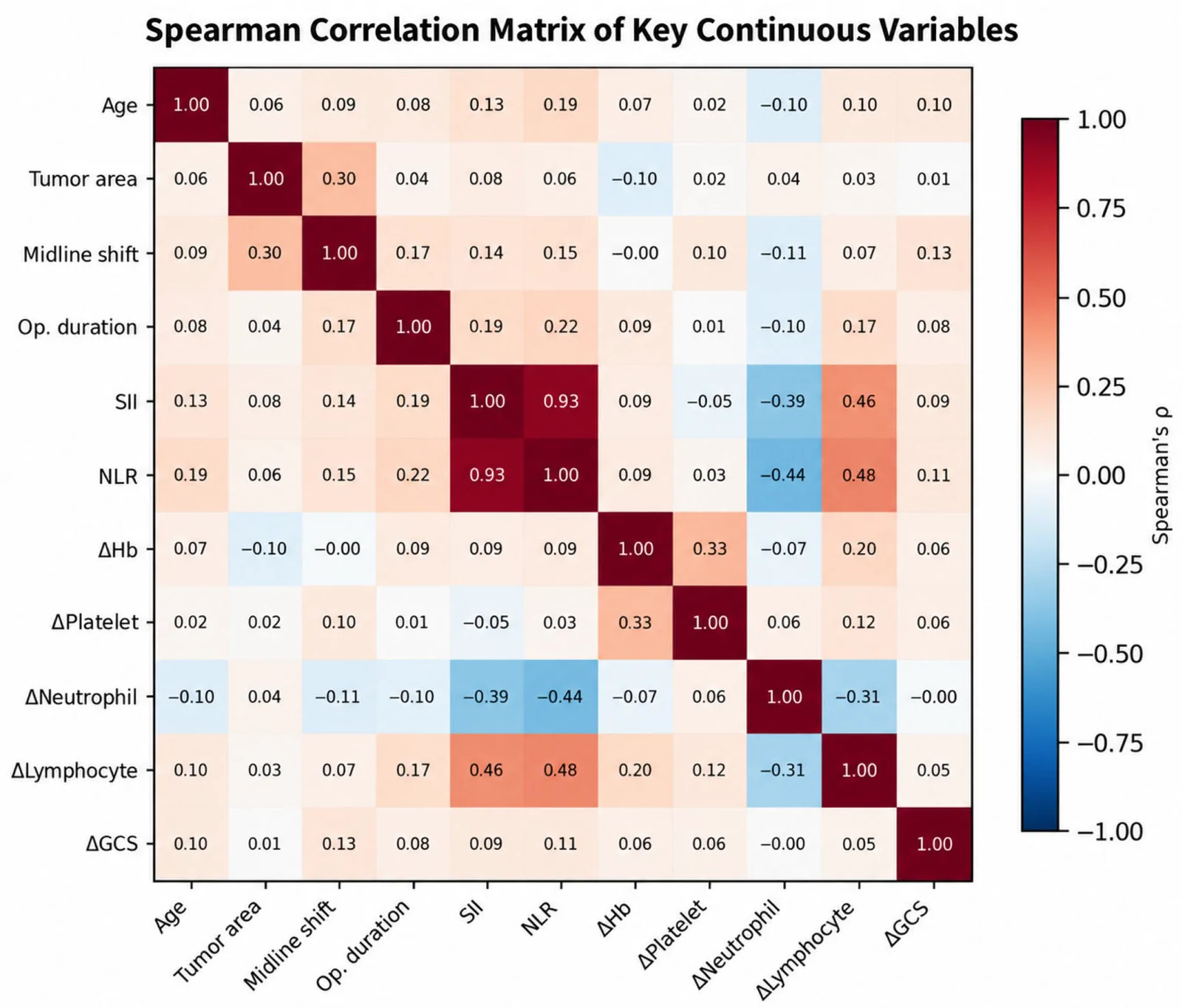
Spearman correlation matrix of key continuous variables.

Lesion area showed a weak-to-moderate positive correlation with midline shift (Spearman’s ρ = 0.303, *p* < 0.001). The correlation matrix for the principal continuous variables is presented in Figure 4, demonstrating a strong correlation between SII and NLR (ρ = 0.93).

### 3.4. Lesion Category and Early Neurological Decompensation

**Figure 5:**
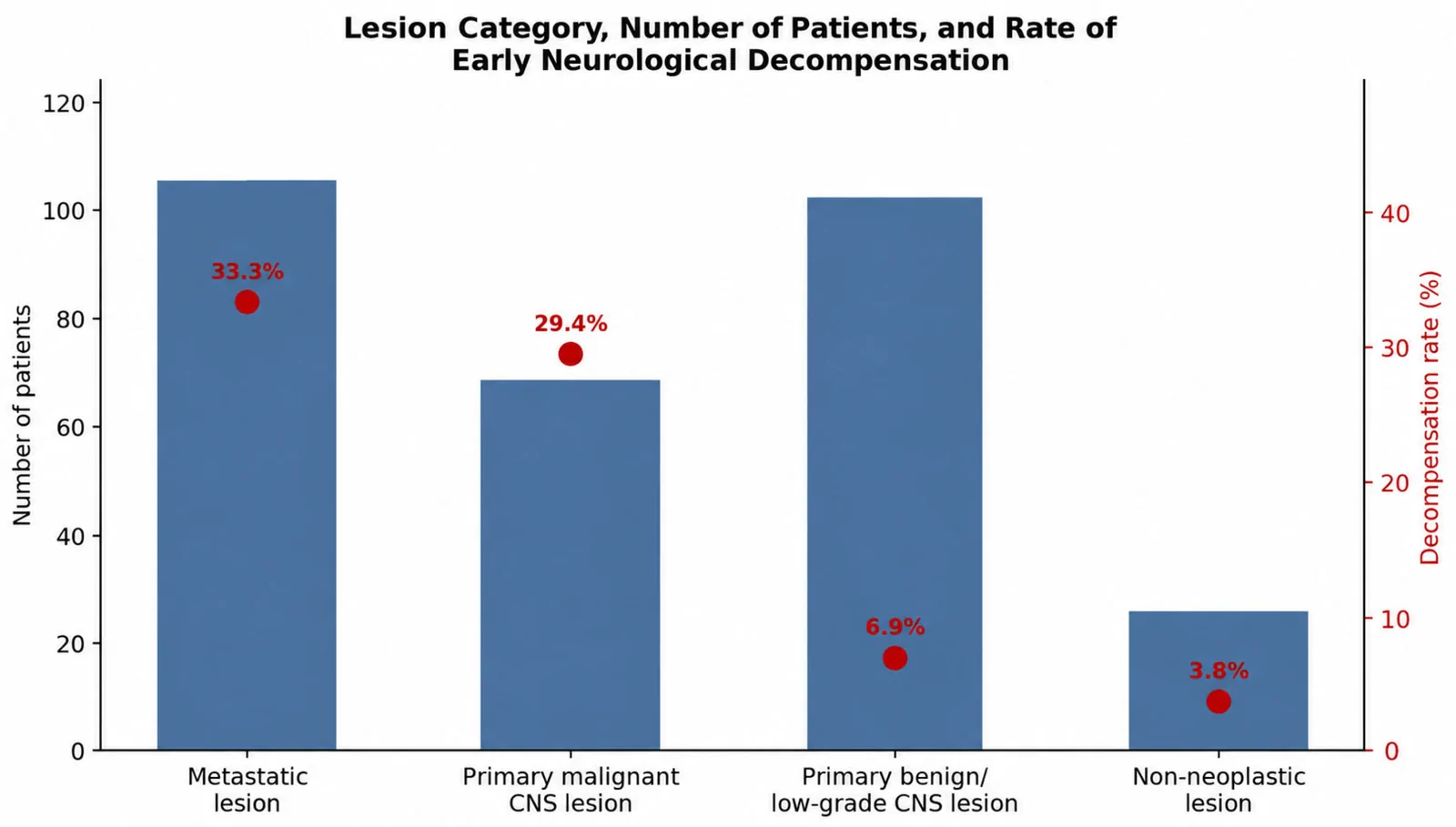
Lesion category, number of surgical episodes, and rate of early neurological decompensation.

Early neurological decompensation differed significantly across lesion categories (χ^2^, *p* < 0.001; Table 1, Figure 5). The highest event rates were observed among metastatic tumors (33.3%, following reclassification of five originally indeterminate lesions as metastatic; see Section 2.3) and primary malignant CNS tumors (29.4%), whereas substantially lower rates were recorded for primary benign/low-grade CNS tumors (6.9%) and non-neoplastic lesions (3.8%). Of the 301 surgical episodes, 266 (88.4%) involved supratentorial lesions and 35 (11.6%) involved infratentorial/posterior fossa lesions; the early neurological decompensation rate was 21.4% (57/266) in the supratentorial group versus 17.1% (6/35) in the infratentorial/posterior fossa group.

### 3.5. Univariable Factors Associated with Early Neurological Decompensation

Univariable logistic regression identified several factors associated with early neurological decompensation (Table 3). Higher age was associated with greater odds of the outcome (OR = 1.04, 95% CI [1.01, 1.06], *p* = 0.002), whereas higher preoperative GCS (OR = 0.74, 95% CI [0.61, 0.90], *p* = 0.003) and KPS (OR = 0.92, 95% CI [0.90, 0.95] per 1-point increase; OR = 0.46, 95% CI [0.35, 0.59] per 10-point increase; *p* < 0.001) were associated with lower odds.

Among inflammatory and perioperative hematological measures, NLR (OR = 1.03, 95% CI [1.01, 1.05], *p* = 0.005), Δneutrophil (OR = 0.93, 95% CI [0.88, 0.99], *p* = 0.031), and Δlymphocyte (OR = 1.45, 95% CI [1.01, 2.08], *p* = 0.043) showed significant associations. In additional baseline-adjusted analyses, the association of Δneutrophil remained statistically significant after adjustment for preoperative neutrophil count (aOR = 0.930, 95% CI [0.868, 0.997], *p* = 0.042). In contrast, the association of Δlymphocyte was attenuated and was no longer statistically significant after adjustment for preoperative lymphocyte count (aOR = 1.121, 95% CI [0.701, 1.792], *p* = 0.633), whereas preoperative lymphocyte count itself was significantly associated with the outcome (aOR = 0.509, *p* = 0.009).

Peritumoral edema was likewise associated with the outcome (OR = 7.11, 95% CI [1.68, 30.18], *p* = 0.008). With homogeneous enhancement as the reference pattern, heterogeneous enhancement (OR = 2.36, 95% CI [1.10, 5.07], *p* = 0.028) and peripheral enhancement (OR = 4.60, 95% CI [1.97, 10.73], *p* < 0.001) were associated with higher odds of early neurological decompensation.

SII (OR = 1.00, 95% CI [1.00, 1.00], *p* = 0.089) and comorbidity (OR = 1.87, 95% CI [0.98, 3.59], *p* = 0.059) did not reach statistical significance. To characterize location more fully, all recorded anatomical locations were consolidated into a reproducible set of categories: frontal, temporal, parietal, frontoparietal, occipital, cerebellar/posterior fossa (reference), and a pooled other/deep-location category comprising skull base, intraventricular, frontotemporal, corpus callosum/thalamic, and temporo-occipital locations. The latter locations were pooled because of the small numbers of surgical episodes and/or outcome events within the individual categories, precluding reliable independent estimates. Univariable ORs for each category relative to the cerebellar/posterior fossa reference are reported in the expanded Table 3.

No statistically significant associations were observed for sex, lesion area, midline shift, extent of resection, neuromonitoring, operative duration, Δhemoglobin, or Δplatelet (all *p* > 0.10).

The uninformative appearance of the SII odds ratio (1.00 per unit) reflects the very large numeric scale of SII rather than an absence of association. On a natural-log scale, SII was associated with the outcome (OR = 1.48, 95% CI [1.12, 1.97], *p* = 0.006 per log-unit; OR = 1.24, 95% CI [0.97, 1.58], *p* = 0.089 per SD). NLR showed a similar pattern (log-scale OR = 1.67, 95% CI [1.22, 2.29], *p* = 0.001; per-SD OR = 1.45, 95% CI [1.12, 1.88], *p* = 0.005).

### 3.6. Multivariable Analysis

**Figure 6:**
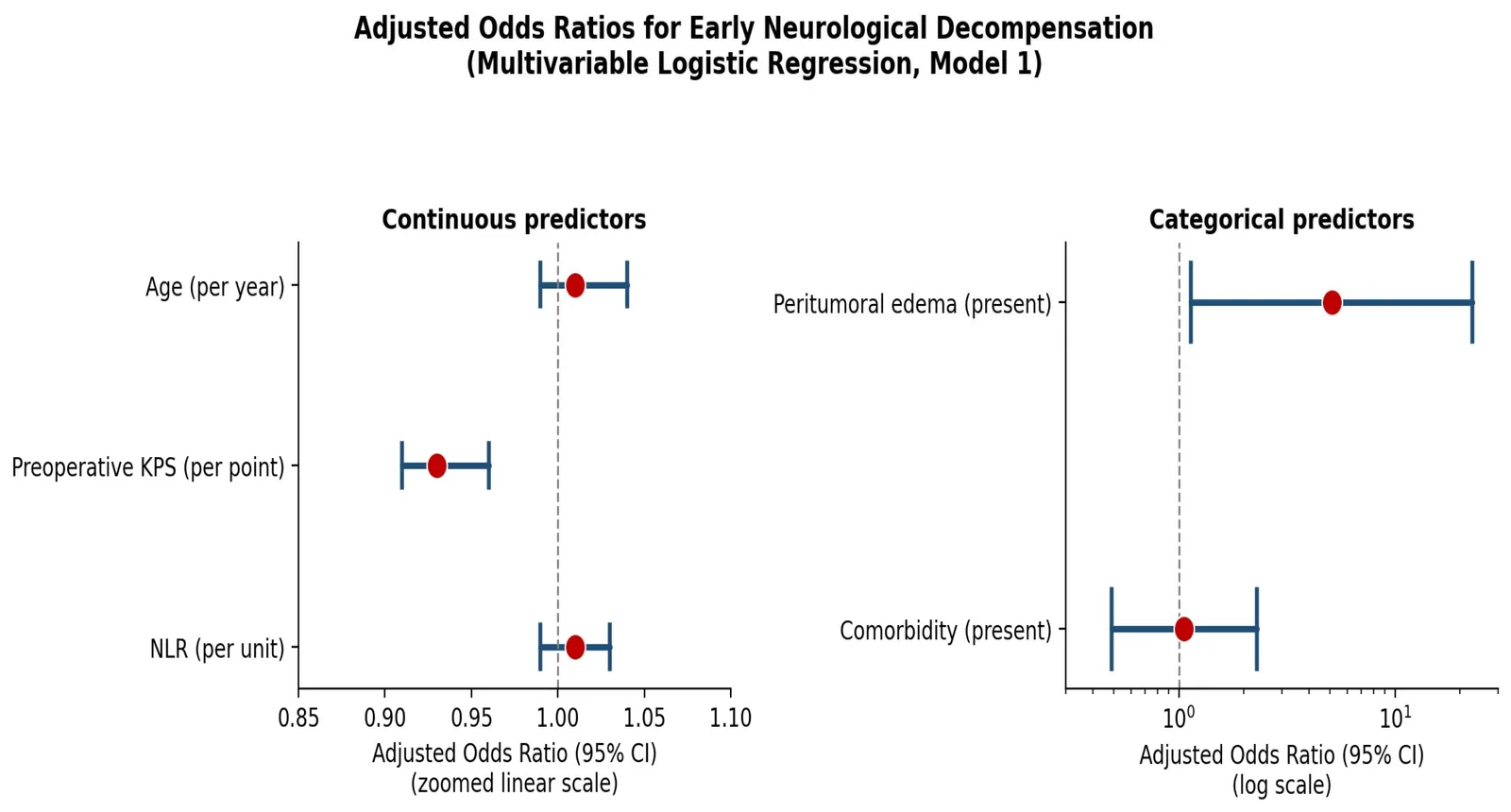
Adjusted odds ratios (95% CIs) from the multivariable logistic regression model. Continuous predictors are displayed on a linear scale and categorical predictors on a logarithmic scale. The dashed vertical line represents the null-effect reference line (OR = 1.0).

The primary multivariable logistic regression model incorporated age, preoperative KPS, peritumoral edema, NLR, and comorbidity (N = 301; Table 4, Figure 6). The model was significant overall (likelihood-ratio test, *p* < 0.001), with a McFadden pseudo-R^2^ of 0.169 and an AUC of 0.793, indicating acceptable discrimination. Variance inflation factors ranged from 1.05 to 1.39, providing no evidence of meaningful multicollinearity.

Following simultaneous adjustment, preoperative KPS (aOR = 0.93, 95% CI [0.91, 0.96], *p* < 0.001 per 1-point increase) remained independently associated with early neurological decompensation, whereas the association with peritumoral edema was statistically significant but imprecisely estimated (aOR = 5.10, 95% CI [1.14, 22.79], *p* = 0.033). In contrast, age (aOR = 1.01, 95% CI [0.99, 1.04], *p* = 0.380), NLR (aOR = 1.01, 95% CI [0.99, 1.03], *p* = 0.478), and comorbidity (aOR = 1.06, 95% CI [0.49, 2.29], *p* = 0.878) were no longer statistically significant after adjustment. For clinical interpretability, the KPS estimate was additionally expressed per 10-point increment: a 10-point higher preoperative KPS was associated with approximately half the odds of early neurological decompensation (aOR = 0.50, 95% CI [0.38, 0.67]).

A quadratic term for preoperative KPS was statistically significant when added to the univariable model (*p* = 0.006), suggesting departure from strict log-linearity. In contrast, quadratic terms for age and NLR were not statistically significant (*p* = 0.639 and *p* = 0.132, respectively).

Preoperative KPS was additionally evaluated as a three-level categorical variable (≤70, 80, and 90–100 [reference]), with adjustment for age, peritumoral edema, NLR, and comorbidity. A graded association was observed: compared with KPS 90–100, KPS 80 was associated with an aOR of 3.32 (95% CI [1.57, 7.03], *p* = 0.002), whereas KPS ≤ 70 was associated with an aOR of 7.15 (95% CI [3.16, 16.20], *p* < 0.001). The categorical model yielded an AUC of 0.779, compared with 0.793 for the primary continuous-KPS model.

Preoperative KPS was also modeled using a restricted cubic spline (3 degrees of freedom) in place of the linear term. A likelihood-ratio test comparing the spline and linear specifications indicated statistically significant non-linearity (χ^2^ = 5.82, df = 1, *p* = 0.016), consistent with the quadratic-term analysis. However, model discrimination was unchanged (AUC = 0.793 for both specifications), suggesting that accounting for non-linearity did not materially improve discrimination.

To evaluate the influence of anatomical location on the KPS association, Model 1 was re-estimated with anatomical location included as a categorical covariate, using cerebellar/posterior fossa as the reference category. The adjusted association of preoperative KPS was essentially unchanged (aOR = 0.929 per 1-point increase, 95% CI [0.901, 0.957], *p* < 0.001, compared with an aOR of 0.93 in the primary model). In this location-adjusted model, frontoparietal location was associated with higher odds of early neurological decompensation relative to the cerebellar/posterior fossa reference category (aOR = 5.02, 95% CI [1.17, 21.47], *p* = 0.029). The estimate for occipital location was elevated but imprecise and was not statistically significant (aOR = 2.77, 95% CI [0.63, 12.12], *p* = 0.177). The remaining anatomical-location categories were not statistically significant. Location-specific estimates should be interpreted cautiously because of the limited number of events in several categories and the resulting wide confidence intervals.

### 3.7. Extended Multivariable Models: Adjustment for Lesion Category and Contrast Pattern

Given the strong relationship between lesion category and early neurological decompensation observed in the unadjusted analysis (Table 1), two extended models were constructed to examine whether the associations of preoperative KPS and peritumoral edema persisted after additional adjustment. In Model 2, the four lesion categories were consolidated into a binary classification: malignant, comprising metastatic and primary malignant CNS tumors (n = 173, 55 events), and non-malignant, comprising primary benign/low-grade CNS tumors and non-neoplastic lesions (n = 128, 8 events). This grouping was used both to maintain an adequate events-per-variable ratio and to reflect the distribution of decompensation rates across lesion categories (Figure 5).

After incorporation of lesion category into the primary model (Table 5, Model 2), malignant lesion category showed an independent association with early neurological decompensation (aOR = 3.76, 95% CI [1.63–8.64], *p* = 0.002), while model discrimination increased to an AUC of 0.814. The estimate for preoperative KPS remained largely unchanged and statistically significant (aOR = 0.94, 95% CI [0.91–0.97], *p* < 0.001). By contrast, the association with peritumoral edema was attenuated and no longer reached conventional statistical significance (aOR = 4.26, 95% CI [0.92–19.73], *p* = 0.064). This attenuation suggests that the association observed for peritumoral edema may partly overlap with lesion-related characteristics represented by lesion category in the extended model.

For Model 3, the four-level contrast-enhancement pattern was incorporated in place of lesion category, with homogeneous enhancement serving as the reference category. None of the patients with non-enhancing lesions (n = 10) experienced early neurological decompensation, resulting in quasi-complete separation for this category. Accordingly, Model 3 was estimated using Firth’s penalized-likelihood logistic regression, as specified in Section 2.6.

In Model 3 (Table 5), peripheral enhancement was independently associated with higher odds of early neurological decompensation compared with homogeneous enhancement (aOR = 3.36, 95% CI [1.36–8.32], *p* = 0.009), whereas neither heterogeneous nor non-enhancing patterns showed significant associations (both *p* > 0.80). Preoperative KPS continued to demonstrate an independent association with the outcome (aOR = 0.93, 95% CI [0.90–0.96], *p* < 0.001). The estimate for peritumoral edema was again attenuated and did not reach conventional statistical significance (aOR = 3.58, 95% CI [0.93–13.79], *p* = 0.064).

**Figure 7:**
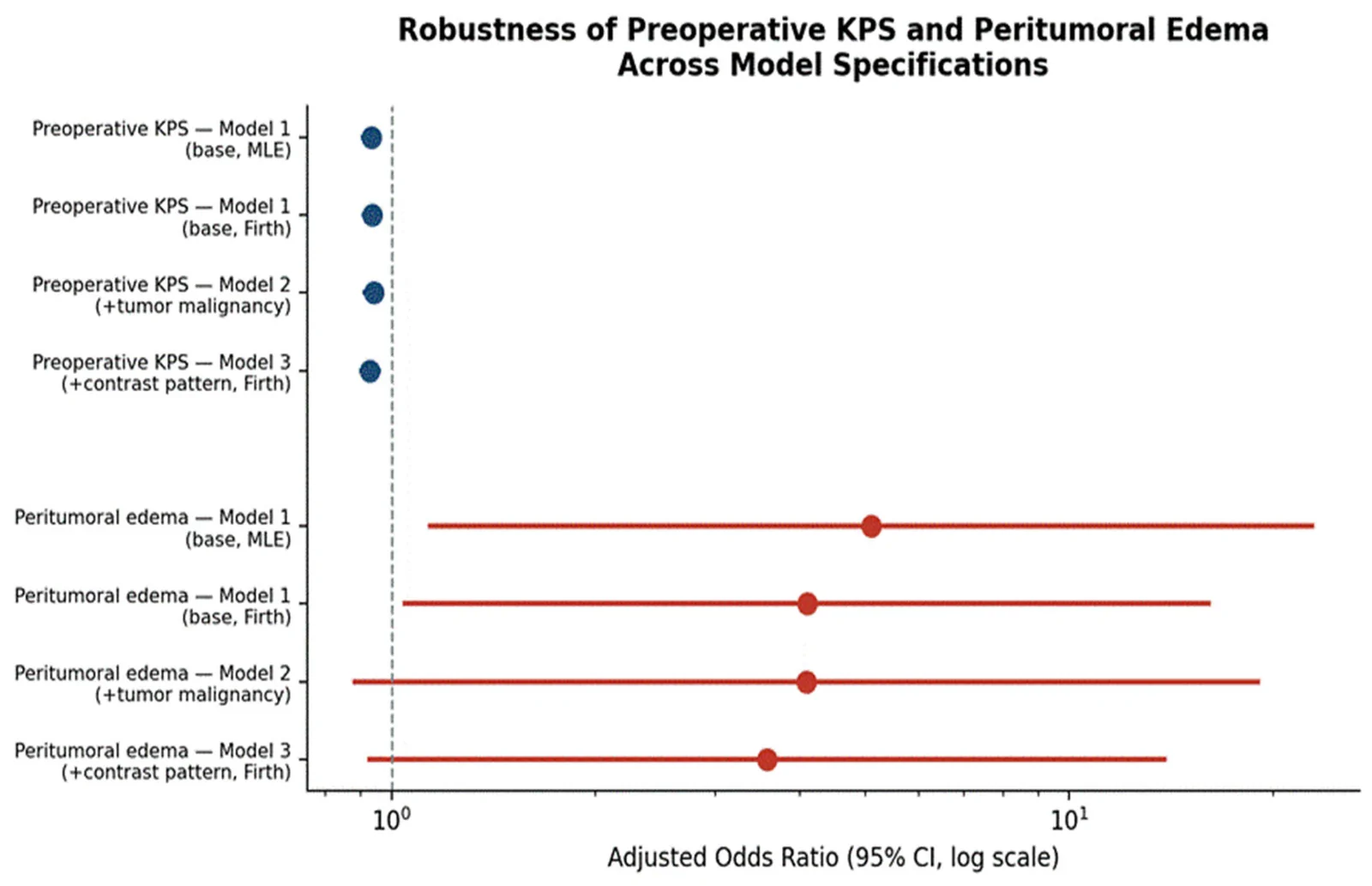
Adjusted odds ratios (95% CI) for preoperative KPS and peritumoral edema across the base model (standard MLE and Firth) and both extended models. The dashed vertical line represents the null-effect reference line (OR = 1.0). Blue indicates preoperative KPS, and red indicates peritumoral edema.

Across the primary and both extended models, preoperative KPS was the only variable to consistently retain a statistically significant independent association with early neurological decompensation (all *p* < 0.001) (Figure 7). Peritumoral edema showed a consistently elevated odds ratio across all three models; however, its association weakened after adjustment for lesion category or contrast-enhancement pattern and no longer reached conventional statistical significance. These findings warrant cautious interpretation of the independent association observed for peritumoral edema in the primary model, as part of this relationship may overlap with lesion-related characteristics.

### 3.8. Firth Penalized Logistic Regression (Robustness Check)

Given the limited number of outcome events (n = 63) and the wide confidence interval surrounding the peritumoral edema estimate in Model 1, the primary model was re-estimated using Firth’s penalized-likelihood logistic regression to assess the robustness of the standard maximum-likelihood estimates. The resulting estimates were consistent with those of the primary analysis (Table 6).

Preoperative KPS retained an independent association with early neurological decompensation (aOR = 0.94, 95% CI [0.91–0.96], *p* < 0.001), while the association with peritumoral edema persisted but remained statistically unstable (aOR = 4.10, 95% CI [1.05–16.03], *p* = 0.043). The direction and magnitude of the penalized estimates were broadly consistent with those of the primary model.

### 3.9. Diagnostic Performance (ROC Analysis)

**Figure 8:**
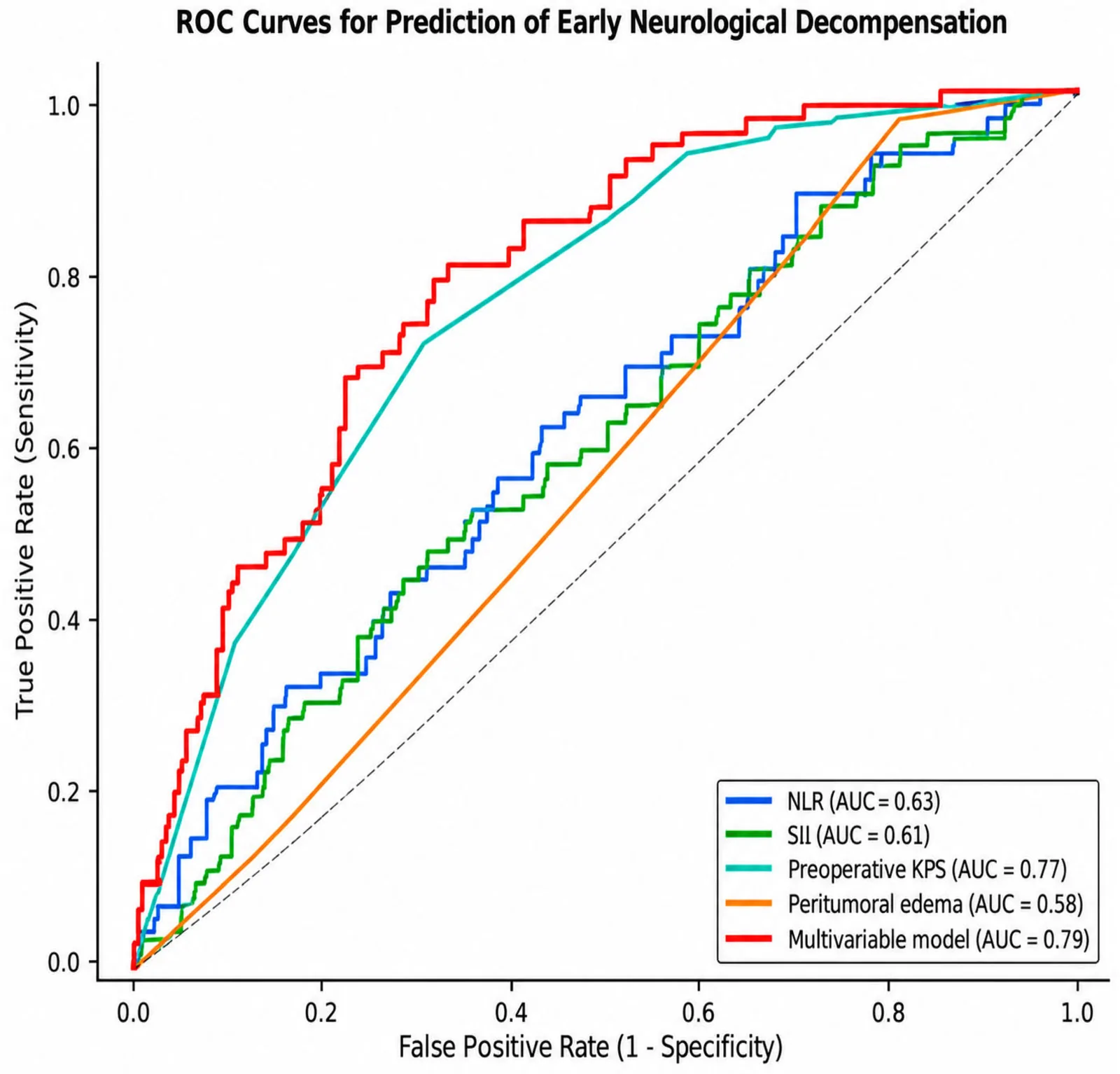
ROC curves for NLR, SII, preoperative KPS, peritumoral edema, and the multivariable model in predicting early neurological decompensation. The dashed diagonal line represents the no-discrimination reference line (AUC = 0.50).

Discriminative performance was examined for the two inflammatory indices of primary interest, NLR and SII, as well as for preoperative KPS and peritumoral edema, the two variables independently associated with early neurological decompensation in the primary multivariable model (Figure 8). Their individual performance was then considered alongside that of the combined model. Lesion area and midline shift were not included in the ROC evaluation because neither demonstrated a significant univariable association with the outcome (Table 1) or entered the multivariable model.

When evaluated individually, NLR yielded an AUC of 0.63 (exploratory Youden-derived cutoff = 9.6, sensitivity = 63%, specificity = 58%), while SII yielded an AUC of 0.61 (exploratory Youden-derived cutoff = 2702, sensitivity = 54%, specificity = 66%), indicating modest discriminatory ability for both inflammatory indices. Preoperative KPS provided substantially greater discrimination with an AUC of 0.772 (95% CI 0.710–0.829; exploratory Youden-derived cutoff = 80, sensitivity = 71%, specificity = 71%), approaching the performance of the full multivariable model. Peritumoral edema, being binary, provided a single meaningful operating point and had limited discrimination when considered alone (AUC = 0.58, sensitivity = 97%, specificity = 19%). Thus, despite showing an independent but statistically imprecise adjusted association in the primary multivariable model, peritumoral edema had limited standalone discriminatory capacity, underscoring the distinction between the magnitude of an adjusted association and the ability of an individual variable to discriminate between outcome groups.

The combined multivariable model yielded the highest point-estimate AUC of 0.793 (95% CI 0.739–0.855), with an exploratory Youden-derived predicted-probability cutoff of 0.18, sensitivity of 81%, and specificity of 68%, compared with preoperative KPS alone or either inflammatory index.

### 3.10. Comparing Model Discrimination: Does Adding Predictors to KPS Improve Prediction?

**Figure 9:**
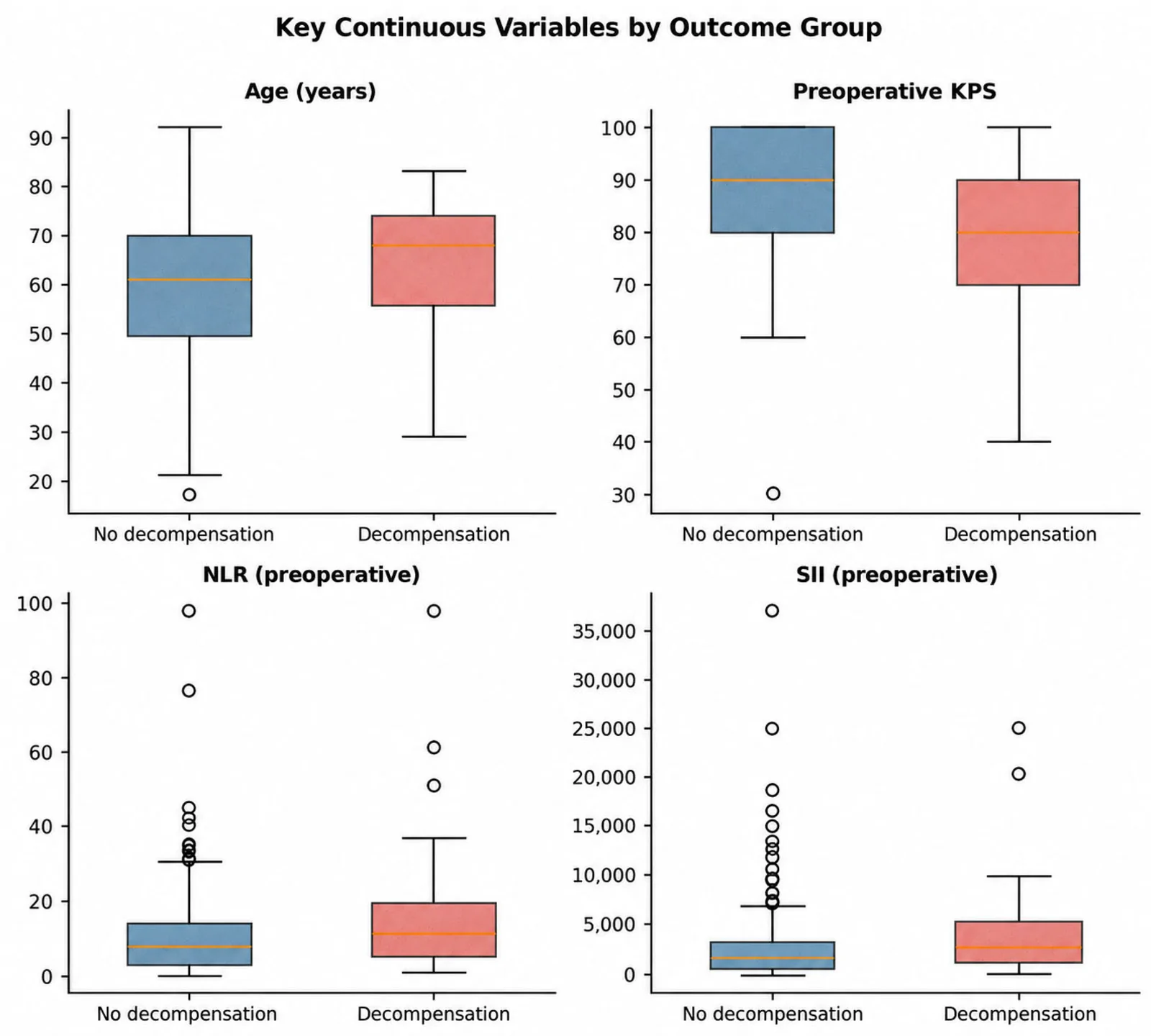
Age, preoperative KPS, NLR, and SII by outcome group.

**Figure 10:**
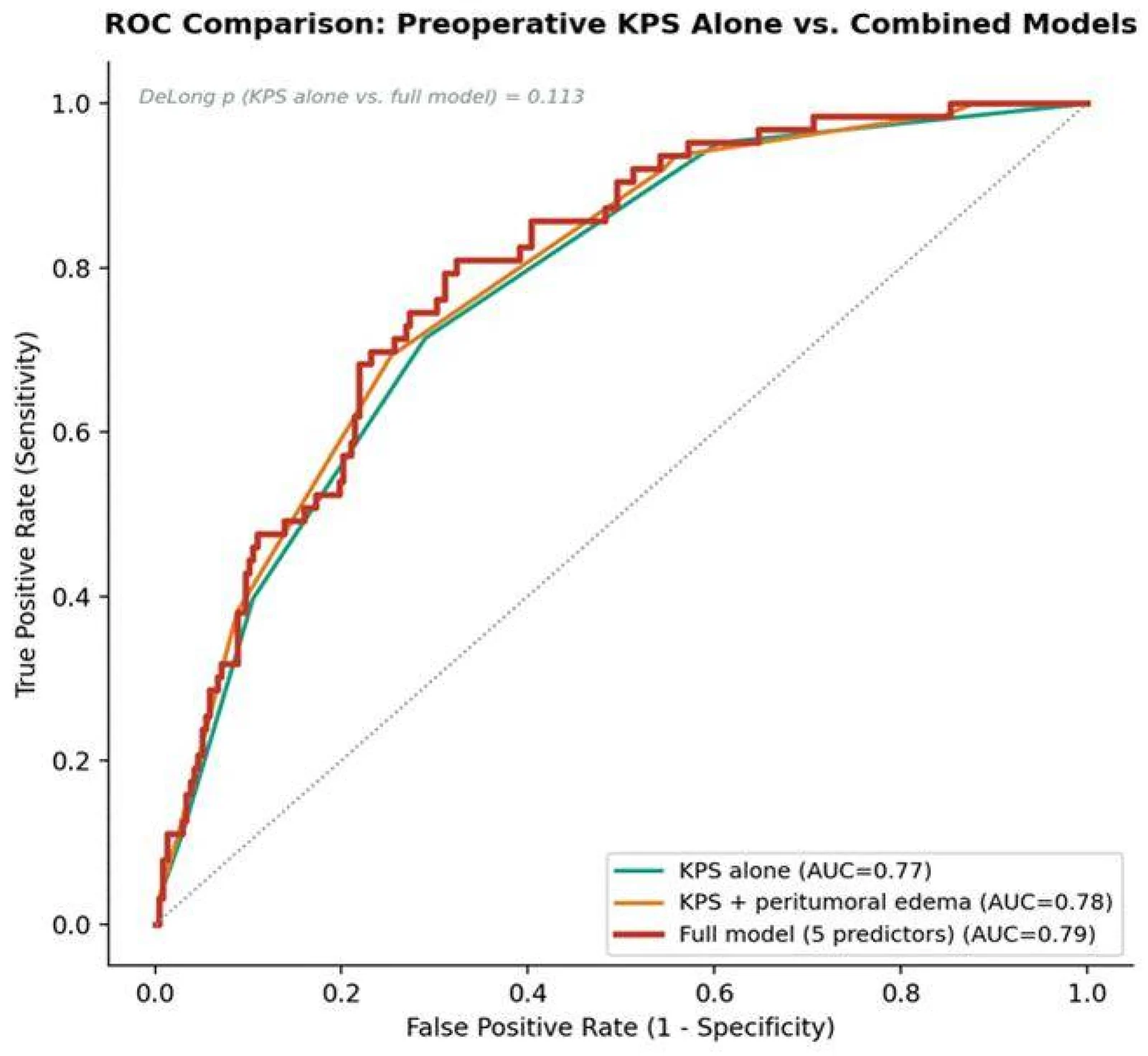
ROC curves for preoperative KPS alone, KPS plus peritumoral edema, and the full base model (N = 301). Bootstrap 95% CIs were obtained using 2000 resamples (percentile method): KPS alone, AUC 0.772 (0.710–0.829), sensitivity 0.714 (0.606–0.981), specificity 0.710 (0.383–0.781), cutoff 0.270; KPS + edema, AUC 0.784 (0.727–0.841), sensitivity 0.698 (0.623–0.971), specificity 0.744 (0.430–0.796), cutoff 0.295; full model, AUC 0.793 (0.739–0.855), sensitivity 0.810 (0.632–0.939), specificity 0.676 (0.536–0.852), cutoff 0.178. DeLong comparisons were nonsignificant (KPS vs. full model, *p* = 0.113; KPS vs. KPS + edema, *p* = 0.365; KPS + edema vs. full model, *p* = 0.289). The dashed diagonal line represents the no-discrimination reference line (AUC = 0.50).

Given the relatively similar discrimination observed for preoperative KPS alone and the full model (Figure 8), the distributions of key continuous variables, including age, preoperative KPS, NLR, and SII, were also examined according to early neurological decompensation status (Figure 9). Incremental model performance was examined using three nested logistic regression models based on the same 301 surgical episodes. The AUC increased from 0.772 for KPS alone to 0.784 after addition of peritumoral edema and to 0.793 for the complete five-predictor model (Figure 10). Pairwise comparisons of the correlated ROC curves using the DeLong test showed no statistically significant AUC differences between KPS alone and KPS plus peritumoral edema (ΔAUC = −0.012, z = −0.90, *p* = 0.365), KPS alone and the full model (ΔAUC = −0.022, z = −1.59, *p* = 0.113), or KPS plus peritumoral edema and the full model (ΔAUC = −0.010, z = −1.06, *p* = 0.289).Thus, although progressively adding predictors produced modest numerical increases in AUC, the present cohort did not demonstrate a statistically significant improvement in discrimination beyond that achieved by preoperative KPS alone. These findings indicate that KPS accounted for a substantial component of the model’s discriminative performance, while the incremental discriminatory contribution of the additional predictors could not be statistically demonstrated in this dataset. For clarity, ΔAUC in Section 3.10 is defined as (AUC of the simpler model − AUC of the more complex model); a negative value therefore denotes a (statistically non-significant) numerical increase in AUC when additional predictors were added to KPS.

### 3.11. Calibration and Internal Validation

**Figure 11:**
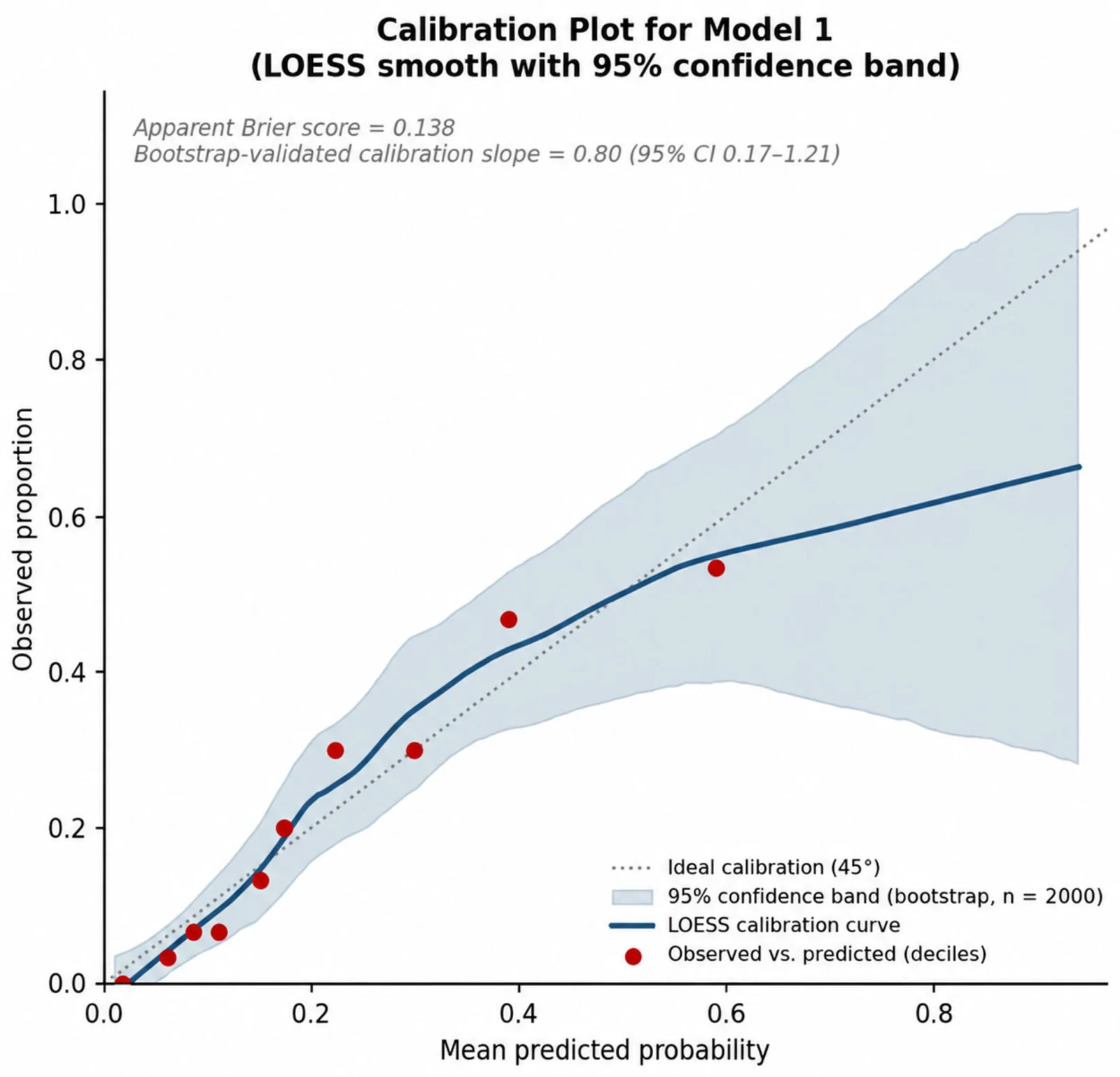
Calibration plot for Model 1 showing decile-based observed versus predicted probabilities, the 45° ideal line, and a LOESS-smoothed calibration curve with a 2000-bootstrap 95% confidence band. The Brier score is also shown.

Calibration of the primary multivariable model (Model 1) was examined using the model’s linear predictor. On the development dataset, the calibration slope was 1.00 (SE = 0.16) and the calibration intercept was 0.00 (SE = 0.24), corresponding to the expected apparent calibration values of 1 and 0, respectively (Figure 11). The model yielded a Brier score of 0.138, compared with 0.165 for the null model based solely on the overall event rate.

To account for optimism inherent in performance estimates obtained from the development sample, internal validation was conducted using 1000 bootstrap resamples and Harrell’s optimism-correction procedure. The model was refitted within each bootstrap sample, and its AUC was evaluated both in the bootstrap sample and in the original development dataset for estimation of optimism. The mean difference between these estimates was used to quantify optimism and was subsequently subtracted from the apparent AUC. The apparent AUC was 0.793 (95% CI [0.739–0.855]), with an estimated optimism of 0.017, resulting in an optimism-corrected AUC of 0.777.

The bootstrap procedure was also used to estimate the internally validated calibration slope. Refitting Model 1 in each of 1000 bootstrap resamples and evaluating the resulting linear predictor in the original development sample yielded an optimism-corrected calibration slope of 0.80 (95% CI [0.17–1.21]), compared with an apparent development-sample slope of 1.00. The wide confidence interval indicates considerable uncertainty in the internally validated calibration estimate. In addition, the LOESS-smoothed calibration curve in Figure 11 suggests overestimation of risk at predicted probabilities above approximately 0.5. Accordingly, absolute risk estimates in this higher range of predicted probability should be interpreted cautiously pending external validation.

### 3.12. Sensitivity Analysis: Repeat-Surgery Episodes

To assess the potential influence of repeated observations, the primary multivariable model was re-estimated after excluding the second surgical episode of the eight patients who underwent repeat operations, resulting in a cohort of 293 unique patients (one surgical episode per patient); this N = 293 cohort is presented as a sensitivity analysis rather than as the primary analysis population, because the repeat-surgery episodes represent a small minority of the cohort (8/301 episodes) and the point estimates were materially unchanged after their exclusion (below). The findings remained consistent with those of the primary analysis. Preoperative KPS retained an independent association with early neurological decompensation (aOR = 0.93, 95% CI [0.91–0.96], *p* < 0.001), as did peritumoral edema (aOR = 5.05, 95% CI [1.10–23.09], *p* = 0.037), whereas age, NLR, and comorbidity remained non-significant. The consistency of these estimates with the primary model indicates that exclusion of repeat-surgery episodes did not materially alter the principal findings (Table 7).

### 3.13. Sensitivity Analysis: Neoplastic Lesions Only

Given the clinical and biological heterogeneity of the cohort, the primary multivariable model was additionally re-estimated in the subset of episodes involving neoplastic lesions after exclusion of the 26 non-neoplastic episodes (N = 275 episodes, 62 events). Preoperative KPS remained independently associated with early neurological decompensation (aOR = 0.93, 95% CI [0.91–0.96], *p* < 0.001). Peritumoral edema was also associated with the outcome, although the estimate remained imprecise (aOR = 5.04, 95% CI [1.12–22.81], *p* = 0.036). Age, NLR, and comorbidity were not independently associated with the outcome. Model discrimination in the neoplastic-only subgroup was similar to that of the full cohort (AUC = 0.786, 95% CI [0.722–0.843], versus AUC = 0.793 in the full cohort). Overall, the principal associations observed in the full cohort were materially similar when the analysis was restricted to neoplastic lesions.

## 4. Discussion

Early neurological decompensation within the first 48 h after intracranial surgery occurred in 20.9% of surgical episodes in this cohort. Preoperative functional status, particularly KPS, showed the most consistent independent association with early neurological decompensation across the multivariable analyses. Although peritumoral edema was independently associated with the outcome in the primary model, this association was attenuated after additional adjustment for lesion category and contrast-enhancement pattern. NLR was associated with the outcome in univariable analysis but did not retain an independent association after multivariable adjustment, and the inflammatory indices alone showed limited discriminatory performance. Importantly, adding peritumoral edema or the full set of Model 1 predictors did not significantly improve discrimination beyond KPS alone. Taken together, these findings identify preoperative functional status as the most consistent factor associated with early neurological decompensation in this cohort, while the additional predictive contribution of the evaluated radiological, lesion-related, and inflammatory variables beyond KPS was limited.

Preoperative functional status emerged as the most consistently associated clinical factor for early neurological decompensation in our cohort. Preoperative KPS retained its independent association across the different multivariable models, with each 10-point increase corresponding to approximately 50% lower odds of early neurological decompensation in the primary model. This observation is consistent with previous evidence supporting the clinical relevance of baseline functional status in patients undergoing intracranial tumor surgery. Among patients treated surgically for glioblastoma, preoperative KPS has been independently associated with early postoperative functional status [11]. More recently, postoperative KPS has also been evaluated as a clinically relevant functional outcome in patients with glioblastoma [6]. Similarly, a large glioblastoma cohort highlighted the importance of functional status and newly developed postoperative neurological deficits in patient outcomes [12]. In our cohort, KPS alone achieved an AUC of 0.772 (95% CI, 0.710–0.829), indicating moderate-to-good discrimination for early neurological decompensation. Importantly, however, adding peritumoral edema or the full set of Model 1 predictors did not significantly improve discrimination beyond KPS alone. These findings support preoperative functional status as the most consistently associated clinical factor in this cohort, while the incremental discriminative contribution of the additional evaluated variables was not demonstrated.

Peritumoral edema was associated with early neurological decompensation and retained an independent association in the primary multivariable model. This observation is consistent with recent evidence linking peritumoral edema to poorer functional outcomes following intracranial tumor surgery [13]. Additional support for the clinical relevance of preoperative edema comes from a large patient series in which its presence was associated with higher rates of postoperative complications and poorer postoperative functional status [14]. The greater frequency of early neurological decompensation among patients with malignant tumors, together with the independent association retained by malignant tumor category after adjustment, further highlights the relevance of tumor pathology to the early postoperative neurological course. In a large-scale analysis of patients undergoing malignant brain tumor resection, postoperative morbidities, including neurological complications, were likewise shown to have important implications for patient outcomes [15]. In the location-adjusted sensitivity analysis, frontoparietal location was also associated with early neurological decompensation (aOR = 5.02, 95% CI [1.17–21.47], *p* = 0.029). However, given the limited number of events within individual anatomical categories and the wide confidence interval, this finding should be interpreted cautiously and considered exploratory.

Peripheral contrast enhancement was also independently associated with early neurological decompensation. This observation is supported by imaging-based evidence indicating that contrast-enhancement characteristics may carry prognostic information in glioblastoma, with ring-like enhancement in particular being associated with less favorable outcomes [16]. Notably, when tumor category or contrast-enhancement pattern was incorporated into the multivariable models, the effect estimate for peritumoral edema remained elevated, although its statistical significance weakened. This pattern suggests that the relationship between edema and early neurological decompensation may partly overlap with characteristics related to the underlying tumor pathology. In contrast, neither lesion area nor midline shift showed a significant association with early neurological decompensation. Considered together, these findings suggest that early postoperative neurological risk was not associated with lesion size or mass effect alone, while associations were observed for peritumoral edema, lesion pathology, and contrast-enhancement characteristics in the respective multivariable models.

Assessment of hematological parameters allowed their associations with early neurological decompensation to be examined alongside clinical, radiological, and lesion-related characteristics. Higher NLR was significantly associated with early neurological decompensation in univariable analysis. Perioperative changes in neutrophil and lymphocyte counts were also associated with the outcome in univariable analyses; however, the directions of these associations did not indicate that greater postoperative neutrophil increases or greater lymphocyte decreases were associated with higher odds of early neurological decompensation. Additional baseline-adjusted analyses further clarified these findings. The association of Δneutrophil persisted after adjustment for preoperative neutrophil count, whereas the association of Δlymphocyte was attenuated and no longer statistically significant after adjustment for its baseline value. These findings suggest that the Δlymphocyte association was substantially influenced by baseline values, whereas the Δneutrophil finding should be interpreted cautiously and requires further investigation. SII did not reach statistical significance in the univariable analysis on its original scale (*p* = 0.089), although an association was observed after logarithmic transformation (*p* = 0.006). Previous evidence has shown significantly higher NLR and SII levels in patients with brain tumors who developed new neurological deficits on the first postoperative day, supporting a potential relationship between systemic inflammatory status and the early postoperative neurological course [17]. Findings from patients undergoing supratentorial brain tumor resection provide further support for this relationship, suggesting that early postoperative NLR and SII levels may be associated with a prolonged need for neurological intensive care [18]. In our cohort, however, NLR did not retain an independent association with early neurological decompensation after multivariable adjustment. Thus, the relationship observed in the univariable analysis did not translate into an independent predictive contribution after clinical and tumor-related factors were considered simultaneously. Taken together, these observations suggest that systemic inflammatory markers may carry information relevant to the early postoperative neurological course, but their predictive contribution should be interpreted within the broader context of clinical and tumor-related characteristics rather than in isolation.

Our findings can also be considered in the context of recent perioperative risk-prediction studies in patients undergoing intracranial tumor surgery. Neumann et al. evaluated the CranioScore and the Munari score in an independent cohort of patients undergoing elective brain tumor resection and reported limited discrimination [19]. These findings highlight the challenges of transporting perioperative risk models across patient populations and clinical settings. In patients undergoing meningioma surgery, Guo et al. developed and validated a nomogram for major postoperative complications that incorporated preoperative KPS, ASA class, tumor characteristics, and perioperative factors [20]. Notably, their definition of major postoperative complications included new neurological deficits, although the overall endpoint was broader than the early neurological decompensation evaluated in our study. More recently, Neumann et al. developed a machine-learning model for adverse events occurring within the first 24 h after elective brain tumor resection, incorporating preoperative and perioperative information [21]. In comparison, our study focused specifically on neurological decompensation within 48 h, and preoperative KPS showed the most consistent independent association across the multivariable analyses, while the addition of the evaluated radiological and inflammatory variables did not significantly improve discrimination beyond KPS alone. Importantly, our study was not designed to establish a clinical risk score. Accordingly, our findings should be viewed as associations requiring external validation rather than as a clinically applicable risk-stratification tool.

A major strength of this study is the evaluation of early neurological decompensation within a unified analytical framework incorporating clinical, radiological, lesion-related, and systemic inflammatory factors. The inclusion of diverse intracranial lesion categories enabled these factors to be evaluated within a broad surgical cohort. The primary multivariable model demonstrated acceptable discrimination, which was largely preserved after bootstrap-based internal validation. Furthermore, the principal associations were similar in the Firth penalized regression and in the sensitivity analysis restricted to one surgical episode per patient.

Several limitations should be considered. The retrospective, single-center design and team-based case selection may introduce selection bias, limit representativeness, and leave residual confounding. The limited number of early neurological decompensation events warrants caution when interpreting subgroup analyses and extended models. All patients received preoperative dexamethasone (8 mg), but the duration of corticosteroid exposure was not consistently available. Its potential effects on neutrophil and lymphocyte counts should therefore be considered when interpreting NLR, SII, and other hematological indices. Although adjustment for anatomical location did not materially alter the association between preoperative KPS and early neurological decompensation, lesion eloquence was not formally classified. Frontoparietal location was associated with the outcome in the location-adjusted sensitivity analysis; however, the limited number of events within anatomical categories and the wide confidence interval warrant caution. These location-specific findings should be considered exploratory. Preoperative KPS, postoperative neurological status, and preoperative radiological assessments were performed by the same neurosurgeon, who was a co-author of the study. The absence of blinding may have introduced assessment bias, particularly when identifying newly developed or objectively worsened neurological deficits. Although motor strength was routinely assessed using the Medical Research Council (MRC) scale, individual preoperative-to-postoperative changes in MRC grade were not available as structured quantitative data, precluding reliable assessment of the magnitude of motor deterioration. Expected versus unexpected postoperative deficits, including supplementary motor area-related syndromes, were not systematically classified. Furthermore, similar group-level preoperative and postoperative KPS medians do not establish that individual deficits were mild or transient. Although no postoperative hematoma or ischemic lesion was identified on early postoperative CT among patients meeting the primary endpoint, CT performed within the first 6 h may not detect all acute ischemic changes. Postoperative edema progression was also not systematically quantified. The term “early neurological decompensation” denotes changes meeting the predefined GCS- or neurological deficit-based criteria within 48 h and does not necessarily imply severe or irreversible deterioration. The cohort included metastatic, primary malignant, benign/low-grade, and non-neoplastic lesions with differing biological and surgical characteristics. Although the neoplastic-only sensitivity analysis yielded materially similar principal associations, the findings may not be generalizable to individual lesion categories. Potentially relevant perioperative factors, including antithrombotic therapy, intraoperative complications, and postoperative ICU management, were not standardized in the multivariable analyses. Lesion size was estimated using a two-dimensional area measure, peritumoral edema was classified dichotomously rather than volumetrically, and comorbidity was recorded as a binary variable rather than using a validated index. Finally, the primary analysis was conducted at the surgical-episode level, including eight patients with repeat procedures. Although the sensitivity analysis restricted to one procedure per patient yielded materially similar findings, within-patient dependence cannot be completely excluded. The wide confidence interval of the optimism-corrected calibration slope and the tendency toward risk overestimation at predicted probabilities above approximately 0.5 indicate uncertainty in the model’s absolute risk estimates. The absence of external validation further limits the generalizability of model performance, and the findings should not be interpreted as establishing a clinically applicable risk-prediction tool.

## 5. Conclusions

This study evaluated multiple clinical, radiological, lesion-related, and hematological factors associated with early neurological decompensation following intracranial mass lesion surgery. Preoperative functional status, as measured by KPS, showed the most consistent independent association with early neurological decompensation. Although peritumoral edema and lesion-related characteristics were associated with the outcome in some multivariable models, their incremental discriminative contribution beyond KPS alone was not demonstrated. NLR was associated with early neurological decompensation in univariable analysis but did not retain an independent association after multivariable adjustment. These findings should be interpreted as associations rather than as an established clinical risk-prediction framework, and external validation in independent, preferably multicenter cohorts is required before clinical application.

## Figures and Tables

**Table 1:** Clinical, radiological, lesion-related, surgical, and laboratory characteristics according to early neurological decompensation status.

Variable	Overall (N = 301)	No Decompensation (n = 238)	Decompensation (n = 63)	*p*
Age, years	62.0 (51.0–71.0)	61.0 (49.2–70.0)	68.0 (55.5–74.0)	=0.001
Preoperative GCS	15.0 (15.0–15.0)	15.0 (15.0–15.0)	15.0 (14.0–15.0)	=0.002
Postoperative GCS	15.0 (15.0–15.0)	15.0 (15.0–15.0)	15.0 (15.0–15.0)	<0.001
Preoperative KPS	90.0 (80.0–100.0)	90.0 (80.0–100.0)	80.0 (70.0–90.0)	<0.001
Postoperative KPS	90.0 (80.0–100.0)	90.0 (90.0–100.0)	80.0 (75.0–90.0)	<0.001
Lesion area, cm^2^	10.3 (5.8–19.3)	10.2 (6.0–19.0)	10.8 (5.3–22.0)	=0.710
Midline shift, mm	0.0 (0.0–2.0)	0.0 (0.0–2.0)	0.0 (0.0–2.0)	=0.561
Operative duration, min	215.0 (180.0–255.0)	215.0 (180.0–258.8)	205.0 (180.0–250.0)	=0.975
Preoperative hemoglobin, g/dL	12.7 ± 1.9	12.8 ± 1.9	12.0 ± 2.1	=0.004
Postoperative hemoglobin, g/dL	12.0 ± 1.8	12.2 ± 1.8	11.2 ± 1.8	<0.001
Preoperative platelet, ×10^9^/L	257.0 (203.0–314.0)	257.5 (208.0–315.5)	257.0 (183.0–309.5)	=0.715
Postoperative platelet, ×10^9^/L	247.0 (191.0–310.0)	247.0 (192.2–301.5)	249.0 (181.5–330.5)	=0.953
Preoperative neutrophil, ×10^9^/L	8.1 (5.2–12.0)	8.1 (5.2–12.0)	7.9 (5.8–12.4)	=0.746
Postoperative neutrophil, ×10^9^/L	10.6 (7.5–14.4)	11.0 (7.7–14.4)	9.3 (6.4–14.2)	=0.140
Preoperative lymphocyte, ×10^9^/L	1.1 (0.7–1.7)	1.1 (0.8–1.8)	0.8 (0.4–1.4)	<0.001
Postoperative lymphocyte, ×10^9^/L	0.8 (0.6–1.2)	0.8 (0.6–1.3)	0.7 (0.5–1.2)	=0.039
SII (preoperative)	2039.7 (939.4–3858.4)	1849.6 (849.0–3394.7)	2832.8 (1349.4–5517.1)	=0.005
NLR (preoperative)	8.5 (3.4–15.0)	7.9 (3.1–14.2)	11.5 (5.2–19.7)	=0.002
Sex, n (%)—*p* = 0.676				
Male	153 (50.8%)	119 (50.0%)	34 (54.0%)	
Female	148 (49.2%)	119 (50.0%)	29 (46.0%)	
Comorbidity, n (%)—*p* = 0.079				
Present	204 (67.8%)	155 (65.1%)	49 (77.8%)	
Absent	97 (32.2%)	83 (34.9%)	14 (22.2%)	
Peritumoral edema, n (%)—*p* = 0.001				
Present	254 (84.4%)	193 (81.1%)	61 (96.8%)	
Absent	47 (15.6%)	45 (18.9%)	2 (3.2%)	
Resection degree, n (%)—*p* = 0.741				
Total	281 (93.4%)	221 (92.9%)	60 (95.2%)	
Subtotal	19 (6.3%)	16 (6.7%)	3 (4.8%)	
Biopsy only	1 (0.3%)	1 (0.4%)	0 (0.0%)	
Contrast-enhancement pattern, n (%)—*p* < 0.001				
Homogeneous	91 (30.2%)	81 (34.0%)	10 (15.9%)	
Heterogeneous	142 (47.2%)	110 (46.2%)	32 (50.8%)	
Peripheral	58 (19.3%)	37 (15.5%)	21 (33.3%)	
None	10 (3.3%)	10 (4.2%)	0 (0.0%)	
Neuromonitoring, n (%)—*p* = 0.999				
Used	255 (84.7%)	202 (84.9%)	53 (84.1%)	
Not used	46 (15.3%)	36 (15.1%)	10 (15.9%)	
Lesion category, n (%)—*p* < 0.001				
Metastatic tumor	105 (34.9%)	70 (29.4%)	35 (55.6%)	
Primary malignant CNS tumor	68 (22.6%)	48 (20.2%)	20 (31.7%)	
Primary benign/low-grade CNS tumor	102 (33.9%)	95 (39.9%)	7 (11.1%)	
Non-neoplastic lesion	26 (8.6%)	25 (10.5%)	1 (1.6%)	

Note: Continuous variables are presented as mean ± SD or median (IQR), according to their distribution, and were compared using the independent-samples *t*-test or Mann–Whitney U test, as appropriate. Categorical variables are presented as n (%) and were compared using the chi-square test or Fisher’s exact test, as appropriate.

**Table 2:** Perioperative changes in neurological, functional, and hematological parameters.

Variable	Preoperative	Postoperative	Median Δ	Test	*p*
Hemoglobin, g/dL	12.7 ± 1.9	12.0 ± 1.8	−0.6 g/dL (IQR −1.4 to 0.1)	Wilcoxon signed-rank	<0.001
Platelet count, ×10^9^/L	257.0 (203.0–314.0)	247.0 (191.0–310.0)	−11.0 (IQR −36 to 18) × 10^9^/L	Wilcoxon signed-rank	<0.001
Neutrophil count, ×10^9^/L	8.1 (5.2–12.0)	10.6 (7.5–14.4)	1.27 (IQR −1.08 to 4.80) × 10^9^/L	Wilcoxon signed-rank	<0.001
Lymphocyte count, ×10^9^/L	1.1 (0.7–1.7)	0.8 (0.6–1.2)	−0.15 (IQR −0.63 to 0.13) × 10^9^/L	Wilcoxon signed-rank	<0.001
GCS	15.0 (15.0–15.0)	15.0 (15.0–15.0)	0 (IQR 0 to 0) points	Wilcoxon signed-rank	=0.010
KPS	90.0 (80.0–100.0)	90.0 (80.0–100.0)	0 (IQR 0 to 0) points	Wilcoxon signed-rank	<0.001

Note: Preoperative and postoperative values were compared using the Wilcoxon signed-rank test. Median Δ represents the median patient-level postoperative–preoperative change. Hemoglobin is reported as mean ± SD, whereas platelet, neutrophil, and lymphocyte counts and GCS and KPS scores are reported as median (IQR), according to their distributions. For GCS, identical group-level medians and IQRs do not indicate the absence of individual paired changes; the Wilcoxon signed-rank test reflects the distribution of patient-level paired differences.

**Table 3:** Univariable logistic regression analysis of factors associated with early neurological decompensation.

Predictor	OR	95% CI	*p*
Age (per year)	1.04	1.01–1.06	=0.002
Preoperative GCS (per point)	0.74	0.61–0.90	=0.003
Preoperative KPS (per point)	0.92	0.90–0.95	<0.001
Δ Neutrophil (per unit)	0.93	0.88–0.99	=0.031
Δ Lymphocyte (per unit)	1.45	1.01–2.08	=0.043
SII (log-transformed, per log-unit increase)	1.48	1.12–1.97	=0.006
NLR (per unit)	1.03	1.01–1.05	=0.005
Comorbidity (present vs. absent)	1.87	0.98–3.59	=0.059
Peritumoral edema (present vs. absent)	7.11	1.68–30.18	=0.008
Location: Parietal (vs. cerebellar/posterior fossa)	1.77	0.58–5.42	=0.316
Contrast pattern: Heterogeneous (vs. homogeneous)	2.36	1.10–5.07	=0.028
Contrast pattern: Peripheral (vs. homogeneous)	4.60	1.97–10.73	<0.001
Location: Frontoparietal (vs. cerebellar/posterior fossa)	3.87	1.08–13.90	=0.038
Location: Occipital (vs. cerebellar/posterior fossa)	3.76	1.00–14.10	=0.050
Location: Temporal (vs. cerebellar/posterior fossa)	1.24	0.40–3.90	=0.710
Location: Frontal (vs. cerebellar/posterior fossa)	1.23	0.42–3.65	=0.704
Location: Other/deep location (vs. cerebellar/posterior fossa)	0.62	0.21–1.86	=0.393
Baseline-adjusted models			
Δ Neutrophil, adjusted for preoperative neutrophil (per unit)	0.930	0.868–0.997	=0.042
Δ Lymphocyte, adjusted for preoperative lymphocyte (per unit)	1.121	0.701–1.792	=0.633
Preoperative lymphocyte, in the Δ-lymphocyte-adjusted model (per unit)	0.509	0.303–0.855	=0.009

Note: Unless otherwise indicated, ORs and 95% CIs were obtained from univariable logistic regression analyses. Anatomical-location categories were analyzed within a single model, using cerebellar/posterior fossa as the common reference category. Candidate predictors for the multivariable analysis were selected based on clinical relevance and univariable findings, without applying a fixed statistical significance threshold, as described in Section 2.6.

**Table 4:** Multivariable logistic regression analysis of factors associated with early neurological decompensation.

Predictor	Adjusted OR	95% CI	*p*
Age (per year)	1.01	0.99–1.04	=0.380
Preoperative KPS (per point)	0.93	0.91–0.96	<0.001
Peritumoral edema (present)	5.10	1.14–22.79	=0.033
NLR (per unit)	1.01	0.99–1.03	=0.478
Comorbidity (present)	1.06	0.49–2.29	=0.878
Location-adjusted sensitivity model (Model 1 + anatomical location category; ref = cerebellar/posterior fossa)			
Frontoparietal (vs. cerebellar/posterior fossa)	5.02	1.17–21.47	=0.029
Occipital (vs. cerebellar/posterior fossa)	2.77	0.63–12.12	=0.177
Parietal (vs. cerebellar/posterior fossa)	1.45	0.43–4.93	=0.551
Frontal (vs. cerebellar/posterior fossa)	1.45	0.44–4.83	=0.543
Other/deep location (vs. cerebellar/posterior fossa)	0.66	0.20–2.23	=0.507
Temporal (vs. cerebellar/posterior fossa)	0.54	0.14–2.05	=0.366
Preoperative KPS in this model (per point)	0.929	0.901–0.957	<0.001

Note: Adjusted ORs with 95% CIs are presented. The location-adjusted sensitivity model included all predictors from Model 1 and anatomical location as a categorical covariate, with cerebellar/posterior fossa as the reference category. For clinical interpretation, the corresponding estimate for preoperative KPS per 10-point increase in Model 1 was aOR = 0.50 (95% CI [0.38–0.67]). OR, odds ratio; CI, confidence interval; KPS, Karnofsky Performance Status; NLR, neutrophil-to-lymphocyte ratio.

**Table 5:** Extended multivariable models incorporating lesion category and contrast-enhancement pattern.

Predictor	Model 2: Adjusted OR	95% CI	*p*	Model 3: Adjusted OR (Firth)	95% CI	*p*
Age	1.01	0.98–1.04	=0.554	1.01	0.98–1.03	=0.579
Preoperative KPS	0.94	0.91–0.97	<0.001	0.93	0.90–0.96	<0.001
Peritumoral edema	4.26	0.92–19.73	0.064	3.58	0.93–13.79	=0.064
NLR	1.00	0.98–1.03	=0.705	1.00	0.98–1.03	=0.724
Comorbidity	1.00	0.45–2.20	=0.998	0.92	0.42–1.98	=0.822
Malignant lesion category	3.76	1.63–8.64	=0.002	—	—	—
Contrast: Heterogeneous (vs. homogeneous)	—	—	—	1.08	0.47–2.47	=0.860
Contrast: Peripheral (vs. homogeneous)	—	—	—	3.36	1.36–8.32	=0.009
Contrast: None (vs. homogeneous)	—	—	—	1.29	0.05–32.26	=0.876

Note: Model 2 included lesion category (malignant vs. non-malignant) and was estimated using standard maximum likelihood logistic regression. Model 3 included contrast-enhancement pattern and was estimated using Firth’s penalized logistic regression because of separation resulting from a zero-event category. OR, odds ratio; CI, confidence interval; KPS, Karnofsky Performance Status; NLR, neutrophil-to-lymphocyte ratio.

**Table 6:** Firth penalized logistic regression as a robustness check of the base multivariable model.

Predictor	Adjusted OR (MLE)	95% CI	*p*	Firth aOR	95% CI	*p*
Age (per year)	1.01	0.99–1.04	=0.380	1.01	0.99–1.04	=0.403
Preoperative KPS (per point)	0.93	0.91–0.96	<0.001	0.94	0.91–0.96	<0.001
Peritumoral edema (present)	5.10	1.14–22.79	=0.033	4.10	1.05–16.03	=0.043
NLR (per unit)	1.01	0.99–1.03	=0.478	1.01	0.98–1.03	=0.511
Comorbidity (present)	1.06	0.49–2.29	=0.878	1.05	0.49–2.23	=0.905

Note: The base model was re-estimated using Firth penalized-likelihood logistic regression and compared with the original maximum-likelihood estimates. OR, odds ratio; CI, confidence interval; KPS, Karnofsky Performance Status; NLR, neutrophil-to-lymphocyte ratio.

**Table 7:** Sensitivity analysis of the multivariable model excluding repeat-surgery episodes (N = 293).

Predictor	Adjusted OR	95% CI	*p*
Age (per year)	1.01	0.98–1.03	=0.570
Preoperative KPS (per point)	0.93	0.91–0.96	<0.001
Peritumoral edema (present)	5.05	1.10–23.09	=0.037
NLR (per unit)	1.02	0.99–1.04	=0.191
Comorbidity (present)	1.26	0.56–2.83	=0.583

Note: Adjusted ORs with 95% CIs are presented. Preoperative KPS is reported per 1-point increase, consistent with the original regression model parameterization. OR, odds ratio; CI, confidence interval; KPS, Karnofsky Performance Status; NLR, neutrophil-to-lymphocyte ratio.

## Data Availability

The data that support the findings of this study are available from the corresponding author upon reasonable request.

## Supplementary Materials

The following supporting information can be downloaded at: https://www.mdpi.com/article/10.3390/diagnostics16193240/s1. File S1: TRIPOD 2015 Checklist: Prediction Model Development (Type 1b); File S2: diagnostıc Analysis_Code_PeerReview_Sensitivity_Analyses.py.
