# Supplementary material for: Which Clinical, Radiological, Inflammatory, and Lesion-Related Characteristics Are Associated with Early Neurological Decompensation After Intracranial Mass Lesion Surgery?
Source: Diagnostics (Basel). 2026 Oct 7;16(19):3240. doi: 10.3390/diagnostics16193240 (PMC13664628; doi:10.3390/diagnostics16193240)
Supplement: Supplementary file 1 [file diagnostics-16-03240-s001.zip › supp/diagnostic TRIPOD_Checklist-Supplementary File S1.pdf]

TRIPOD 2015 Checklist: Prediction Model Development (Type 1b)

Manuscript: "Which Clinical, Radiological, Inflammatory, and Lesion-Related Characteristics Are Associated with Early Neurological Decompensation after Intracranial Mass Lesion Surgery?" — Saylak FB, Bahadır B.

TRIPOD type for this study: 1b — a single-cohort, single-model development study without a separate external validation dataset, in which internal validation was performed by bootstrap resampling (1000 resamples, Harrell's optimism-correction method) within the same dataset (Section 2.6, Section 3.11). No external validation cohort was used; consistent with this, the manuscript explicitly states in the Discussion and Conclusion that the reported associations should not be interpreted as an established, clinically applicable risk-prediction tool without external validation. Section/table/figure references below correspond to the section numbering of the resubmitted manuscript version; exact page numbers will be assigned at the production/typesetting stage by the journal.

Section/Topic	Item	Checklist Item	Reported in manuscript (location / details)
**Title and abstract**	**1**	Identify the study as developing and/or validating a multivariable prediction model, the target population, and the outcome to be predicted.	Reported with a caveat. Title identifies the population (patients undergoing intracranial mass lesion surgery), the outcome (early neurological decompensation), and the associative/multivariable nature of the analysis. The manuscript is framed primarily as a risk-factor association study rather than as the development of a named clinical risk score; this framing is stated explicitly in the Discussion (Section 4, "our study was not designed to establish a clinical risk score") and Conclusion (Section 5).
	**2**	Provide a summary of objectives, study design, setting, participants, sample size, predictors, outcome, statistical analysis, results, and conclusions.	Reported. Structured Abstract (Background/Objective, Methods, Results, Conclusions).
**Introduction**	**3a**	Explain the medical context and rationale for developing or validating the multivariable prediction model, including references to existing models.	Reported. Section 1 (Introduction), with references to prior perioperative risk-prediction studies (CranioScore, Munari score, glioma/meningioma nomograms) discussed in Section 4.
	**3b**	Specify the objectives, including whether the study describes the development or validation of the model, or both.	**Partially reported.** Section 1 (Introduction, final paragraph) specifies the objective of identifying factors associated with early neurological decompensation. Section 2.6 describes the development of multivariable logistic regression models and their internal validation using bootstrap resampling. No external validation was performed, and the study was not intended to establish a clinically applicable prediction tool.
**Methods**	**4a**	Describe the study design or source of data (e.g., randomized trial, cohort, or registry data), separately for the development and validation data sets, if applicable.	Reported. Section 2.1: single-center retrospective cohort study; single dataset used for both development and internal (bootstrap) validation.
	**4b**	Specify the key study dates, including start of accrual, end of accrual, and, if applicable, end of follow-up.	Reported. Section 2.1: surgical episodes between 1 April 2019 and 1 January 2026; outcome ascertained within the first 48 h after surgery (Section 2.5).
	**5a**	Specify key elements of the study setting, including number and location of centres and date of restriction, if applicable.	Reported. Section 2.1: single center (Department of Neurosurgery, Ankara Bilkent City Hospital), source population restricted to cases managed by a specific neurosurgical team.
	**5b**	Describe eligibility criteria for participants.	Reported. Section 2.1 and Section 2.1 caption of Figure 1 (study flow diagram); inclusion required age ≥18 years, surgical treatment for an intracranial mass lesion, and availability of preoperative/early postoperative neurological data.
	**5c**	Give details of treatments received, if relevant.	Reported. Section 2.1–2.3: craniotomy approach, extent of resection categories, intraoperative neuromonitoring use; all procedures elective (Section 2.1, as clarified in this revision).
	**6a**	Clearly define the outcome that is predicted by the prediction model, including how and when assessed.	**Reported.** Section 2.5: Early neurological decompensation within 48 h after surgery, defined as a ≥2-point decrease in GCS and/or the development of a new objective neurological deficit or objective worsening of a pre-existing deficit.

	**6b**	Report any actions to blind assessment of the outcome to be predicted.	Reported as a limitation, not as blinding achieved. Section 2.5 and Section 2.2 state explicitly that postoperative assessment was performed by the same, non-blinded assessor (a study co-author); this is discussed as a source of potential assessment bias in the Limitations paragraph of Section 4, added in response to peer review (Comment 8).
	**7a**	Clearly define all predictors used in developing the multivariable prediction model, including how and when they were measured.	Reported. Section 2.2 (clinical/perioperative), Section 2.3 (radiological/lesion-related), Section 2.4 (laboratory/inflammatory indices).
	**7b**	Report any actions to blind assessment of predictors for the outcome and other predictors.	Reported as a limitation. Section 2.2–2.3 and the corresponding Limitations paragraph state that KPS, postoperative neurological status, and radiological measurements were all performed by the same, non-blinded co-author assessor (added in response to peer review, Comment 8).
	**8**	Explain how the study size was arrived at.	Reported as a limitation rather than a pre-specified calculation. Section 2.1 reports the number of surgical episodes assessed for eligibility (n = 360) and excluded (n = 59); no prospective sample-size calculation was performed, consistent with the retrospective design. Section 2.6 explicitly links the choice of a 5-predictor parsimonious primary model to the number of observed outcome events (n = 63) and the resulting events-per-variable ratio.
	**9**	Describe how missing data were handled, with details of any imputation method.	Reported. Section 2.6: no missing values were present for any variable used in the reported analyses; complete-case analysis, no imputation required.
	**10a**	Describe how predictors were handled in the analyses.	Reported. Section 2.6: continuous predictors modelled on their natural scale (with sensitivity analyses for KPS categorization/splines, Section 3.6, added in response to peer review, Comment 5); categorical predictors (lesion location, contrast pattern, lesion category) modelled with dummy coding against a single specified reference category.
	**10b**	Specify type of model, all model-building procedures (including any predictor selection), and method for internal validation.	Reported. Section 2.6: binary logistic regression; Model 1 predictors selected based on clinical relevance and univariable findings, without applying a fixed statistical significance threshold (Table 3), explicitly justifying the exclusion of SII in favour of NLR due to collinearity; two extended models (Model 2 and Model 3, with Firth penalization applied to Model 3) described in Sections 2.6 and 3.7; internal validation via 1000-resample bootstrap optimism correction (Sections 2.6 and 3.11).
	**10c**	For validation, describe how the predictions were calculated.	Not applicable — no separate external validation dataset was used (TRIPOD Type 1b; internal bootstrap validation only, Section 3.11).
	**10d**	Specify all measures used to assess model performance and, if relevant, to compare multiple models.	Reported. Section 2.6 and Section 3.9–3.11: AUC (with 95% CI via bootstrap), sensitivity/specificity at the Youden-optimal cutoff, DeLong test for AUC comparison between nested models, calibration slope, calibration intercept, Brier score, and a LOESS calibration curve with bootstrap confidence band.
	**10e**	Describe any model updating (e.g., recalibration) arising from the validation, if done.	Not applicable — no external validation or recalibration was performed.
	**11**	Provide details on how risk groups were created, if done.	Not applicable. The study did not define clinical risk groups or a scoring/points system; it reports predictor-outcome associations and overall model discrimination/calibration rather than a stratification tool, as stated explicitly in Section 4 (Discussion) and Section 5 (Conclusion).
	**12**	For validation, identify any differences from the development data in setting, eligibility criteria, outcome, and predictors.	Not applicable — single-dataset development study; no separate validation cohort.
**Results**	**13a**	Describe the flow of participants through the study,	Reported. Section 2.1 and Figure 1 (flow diagram: 360

		including the number of participants with and without the outcome.	assessed, 59 excluded, 301 analyzed); Section 3.1 reports 63/301 (20.9%) episodes with the outcome.
	**13b**	Describe the characteristics of the participants (basic demographics, clinical features, available predictors and outcome), including the number of participants with missing data.	Reported. Section 3.1 and Table 1 (baseline characteristics by outcome status); Section 2.6 confirms no missing data.
	**13c**	For validation, show a comparison with the development data of the distribution of important variables.	Not applicable — no separate validation dataset.
	**14a**	Specify the number of participants and outcome events in each analysis.	Reported. Section 3.1 (N = 301, 63 events, primary analysis); Section 3.6/Table 4 report the primary multivariable analysis, while the corrected Table 3 presents the anatomical-location analysis using cerebellar/posterior fossa as the reference category; Section 3.12/Table 7 report the sensitivity analysis excluding repeat surgical observations (N = 293); Section 3.13 reports the neoplastic-only subgroup analysis (N = 275, 62 events).
	**14b**	If done, report the unadjusted association between each candidate predictor and outcome.	Reported. Table 3 (univariable logistic regression for the evaluated candidate predictors, including anatomical location and the log-scale SII estimate).
	**15a**	Present the full prediction model to allow predictions for individuals (i.e., all regression coefficients, and model intercept or baseline survival at a given time point).	Partially reported, by design. Adjusted odds ratios and 95% CIs for all retained predictors in Model 1 and the extended models are reported in Table 4, Table 5, and Table 6; the model intercept/constant is not separately tabulated, consistent with the manuscript's framing as a risk-factor association analysis rather than an individual-patient prediction tool (see item 1 and item 11 above, and Section 4/5).
	**15b**	Explain how to the use the prediction model.	Not applicable, by design. The manuscript explicitly states that it was not intended to yield a clinically applicable prediction tool (Section 4, Section 5); accordingly, no instructions for calculating individual risk scores are provided.
	**16**	Report performance measures (with CIs) for the prediction model.	Reported. Sections 3.9–3.11 and Figure 8: ROC analyses for KPS alone, peritumoral edema alone, NLR, SII, and the combined Model 1, including the AUC with 95% CI for Model 1 (AUC = 0.793, 95% CI 0.739–0.855); Section 3.11: bootstrap optimism-corrected AUC (0.777) and optimism-corrected calibration slope (0.80, 95% CI 0.17–1.21).
	**17**	If done, report the results from any model updating (i.e., model specification, model performance).	Not applicable — no model updating/recalibration was performed.
**Discussion**	**18**	Discuss any limitations of the study (such as nonrepresentative sample, few events per predictor, missing data).	Reported. Section 4, Limitations paragraph: single-center retrospective design, limited number of events, non-blinded single assessor, absence of a validated comorbidity index, absence of external validation, and other items listed explicitly.
	**19a**	For validation, discuss the results with reference to performance in the development data, and any other validation data.	Partially reported. Section 3.11 presents the bootstrap internal-validation results, including the optimism-corrected AUC and calibration slope. These findings and the absence of external validation are discussed in Section 4. No separate external validation dataset was used.
	**19b**	Give an overall interpretation of the results, considering objectives, limitations, results from similar studies, and other relevant evidence.	Reported. Section 4 (Discussion), with explicit comparison to prior perioperative risk-prediction studies (CranioScore, Munari score, meningioma nomogram, ML-based adverse-event model).
	**20**	Discuss the potential clinical use of the model and implications for future research.	Reported. Section 4 and Section 5 (Conclusion): findings framed as hypothesis-generating associations requiring external, preferably multicenter validation before any clinical application; no specific clinical-use pathway is proposed, consistent with the study's stated scope.

**Other information**	**21**	Provide information about the availability of supplementary resources, such as study protocol, Web calculator, and data sets.	Reported. Data Availability Statement (data available from the corresponding author on reasonable request); the completed TRIPOD checklist (Supplementary File S1) and the analysis code (Supplementary File S2) are provided as supplementary material.
	**22**	Give the source of funding and the role of the funders for the present study.	Reported. Funding statement: "This research received no external funding."

Prepared by the authors for this revision, following the TRIPOD 2015 checklist for multivariable prediction model development (type 1b). See also: Collins GS, Reitsma JB, Altman DG, Moons KG. Transparent Reporting of a multivariable prediction model for Individual Prognosis Or Diagnosis (TRIPOD): the TRIPOD statement. BMJ. 2015;350:g7594.
